# Small Molecule NIR‐II Dyes for Switchable Photoluminescence via Host –Guest Complexation and Supramolecular Assembly with Carbon Dots

**DOI:** 10.1002/advs.202202414

**Published:** 2022-06-03

**Authors:** Dinabandhu Sar, Fatemeh Ostadhossein, Parikshit Moitra, Maha Alafeef, Dipanjan Pan

**Affiliations:** ^1^ Bioengineering Department University of Illinois at Urbana‐Champaign Urbana IL 61801 USA; ^2^ Department of Pediatrics Center for Blood Oxygen Transport and Hemostasis University of Maryland Baltimore School of Medicine Health Sciences Research Facility III 670 W Baltimore St. Baltimore MD 21201 USA; ^3^ Department of Chemical Biochemical and Environmental Engineering University of Maryland Baltimore County Interdisciplinary Health Sciences Facility 1000 Hilltop Circle Baltimore MD 21250 USA; ^4^ Biomedical Engineering Department Jordan University of Science and Technology Irbid 22110 Jordan; ^5^ Department of Diagnostic Radiology and Nuclear Medicine University of Maryland Baltimore School of Medicine Health Sciences Research Facility III 670 W Baltimore St. Baltimore MD 21201 USA

**Keywords:** carbon dots, host–guest complexation, NIR‐II emission, supramolecular assembly, switchable fluorescence

## Abstract

Small molecular NIR‐II dyes are highly desirable for various biomedical applications. However, NIR‐II probes are still limited due to the complex synthetic processes and inadequate availability of fluorescent core. Herein, the design and synthesis of three small molecular NIR‐II dyes are reported. These dyes can be excited at 850–915 nm and emitted at 1280–1290 nm with a large stokes shift (≈375 nm). Experimental and computational results indicate a 2:1 preferable host–guest assembly between the cucurbit[8]uril (CB) and dye molecules. Interestingly, the dyes when self‐assembled in presence of CB leads to the formation of nanocubes (≈200 nm) and exhibits marked enhancement in fluorescence emission intensity (Switch‐On). However, the addition of red carbon dots (rCDots, ≈10 nm) quenches the fluorescence of these host–guest complexes (Switch‐Off) providing flexibility in the user‐defined tuning of photoluminescence. The turn‐ON complex found to have comparable quantum yield to the commercially available near‐infrared fluorophore, IR‐26. The aqueous dispersibility, cellular and blood compatibility, and NIR‐II bioimaging capability of the inclusion complexes is also explored. Thus, a switchable fluorescence behavior, driven by host–guest complexation and supramolecular self‐assembly, is demonstrated here for three new NIR‐II dyes.

## Graphical abstract

Three small molecular NIR‐II dyes are employed those exhibit marked enhancement in emission intensity (Switch‐On) when self‐assembled in presence of cucurbit[8]uril. Further addition of red carbon dots to the nanocubes leads to the queching of fluorescence of these host–guest complexes (Switch‐Off) providing flexibility in the user‐defined tuning of photoluminescence.

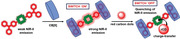

## 1 Introduction

Fluorescence imaging is an important tool in biotechnology‐related investigations and assays as it offers real‐time quantitative monitoring capabilities.^[^ ^1^, ^2^, ^3^, ^4^, ^5^ ^]^ Although in recent years, several near‐infrared window I (NIR‐I) emitting probes (700–900 nm) have been suggested for in vivo imaging, their applications are limited for clinical use due to the low resolution, auto‐fluorescence in that window and poor penetration depth of optical techniques.^[^ ^6^, ^7^, ^8^, ^9^, ^10^ ^]^ Hence, to circumvent some of these shortcomings, biomedical imaging in the second near‐infrared biological window, i.e., 1000–1700 nm, has gained considerable popularity in current years.^[^ ^11^, ^12^, ^13^, ^14^, ^15^, ^16^, ^17^, ^18^ ^]^ Real‐time intraoperative imaging, combined with enhanced depth of penetration, low light scattering, diminished autofluorescence from tissue, high sensitivity and enhanced spatiotemporal resolution are only a few of the merits that NIR‐II imaging can provide especially for in vivo and vasculature studies.^[^ ^19^, ^20^, ^21^, ^22^, ^23^, ^24^, ^25^ ^]^ Successful imaging of the microvasculature,^[^ ^26^, ^27^ ^]^ hemodynamic measurements,^[^ ^28^, ^29^ ^]^ precise control over CRISPR‐Cas9 delivery,^[^ ^30^ ^]^ and molecular imaging of immune checkpoint PD‐L1^[^ ^31^ ^]^ have been demonstrated previously using near infrared biological window II (NIR‐II) probes. Very recently, target sensitivity as high as 77.4 × 10^−15^ m has been reported and the first NIR‐II image‐guided in‐human tumor resection surgery has been demonstrated.^[^ ^32^ ^]^

Although nanomaterials such as quantum dots,^[^ ^33^, ^34^, ^35^ ^]^ carbon nanotubes,^[^ ^36^, ^37^, ^38^ ^]^ and rare‐earth nanoparticles^[^ ^39^, ^40^ ^]^ have pushed the limits of NIR I to NIR III window, there are concerns regarding their biocompatibility compared with small molecule organic fluorophores. On the other hand, the rapid clearance of the dyes makes the targeted imaging practically challenging compared to the nanoparticles.^[^ ^41^ ^]^ Sincere efforts have been expended in the literature in this regard elevating the quantum yield (QY) or Stoke's shift of the current NIR‐II dyes when the dye assembly was embedded in a polymer or protein‐based shells.^[^ ^32^, ^42^, ^43^ ^]^ However, the fluorescence emission of these NIR‐II dyes tend to get quenched due to an effect known as aggregation caused quenching (ACQ) when assembled with different nanoparticles.^[^ ^44^ ^]^ This would impede employment of these dyes in nanoparticle platforms. Hence, we thought of a platform where small molecular organic NIR‐II dyes will supra molecularly self‐assemble into defined nanoparticle shapes with tunable photoluminescence properties. To the best of our knowledge, this kind of approach has not been explored yet for dyes that emit within the second biological window.

Herein, we report a novel photo‐switchable NIR‐II emitting supramolecular system leveraged by the specific interaction of de novo NIR‐II dyes and cucurbit[8]uril (CB[8]) to remarkably enhance the fluorescence of the NIR‐II dyes (turn‐on). The fluorescence is subsequently quenched when red‐emitting CDots are added to the system (turn‐off). The dye synthesized here is based on donor–acceptor–donor (D–A–D) scheme, as has recently been configured for several other NIR‐II dyes.^[^ ^27^, ^43^, ^45^ ^]^ It was presumed that the particular D–A–D design, presented herein, would decrease the bandgap and enhance the conjugation length so as to push the emission toward higher wavelengths.^[^ ^45^ ^]^ Importantly, we observed well‐defined geometrical nanostructures upon the addition of CB[8] to the NIR‐II dyes which may primarily be attributed to the supramolecularly assembled host–guest complexation between the moieties.

Supramolecular assemblies rely on the noncovalent interactions to enable spontaneous construction of ordered nanostructures during the assembly processes.^[^ ^46^ ^]^ On the other hand, charge transfer events often promote unprecedented optical properties triggered by the alternating electron distribution between the supramolecular assembly of host–guest molecules.^[^ ^47^, ^48^ ^]^ In this regard, macrocyclic host molecules, especially cucurbituril, is known to have the potential to self‐assemble with small molecules into sophisticated supramolecular nanoarchitectures.^[^ ^49^, ^50^ ^]^ CB[8] with symmetrical structure, a hydrophobic cavity, and hydrophilic rim, can self‐assemble with a wide variety of guest ligands.^[^ ^51^, ^52^ ^]^ As a result, more diverse supramolecular structures along with tunable fluorescence from its guest chromophore moiety are potentially achievable. This is feasible when the position of the guest molecule changes in the CB[8] scaffold resulting in the variation of its physicochemical properties. Subsequently, this will lead to the change in the emission wavelength of the guest chromophore, while entrapped in the host cavity.^[^ ^52^, ^53^ ^]^ Meanwhile, carbon dots (CDots) are an emerging class of the carbon nanoparticle family and have garnered a lot of attention during the recent times which may be attributed to their extraordinary fluorescent behavior, improved colloidal stability, biocompatibility, and green and scalable synthetic procedures.^[^ ^54^, ^55^, ^56^, ^57^, ^58^ ^]^ We have explored these opportunities in this article.

## 2 Results and Discussion

**Scheme 1:**
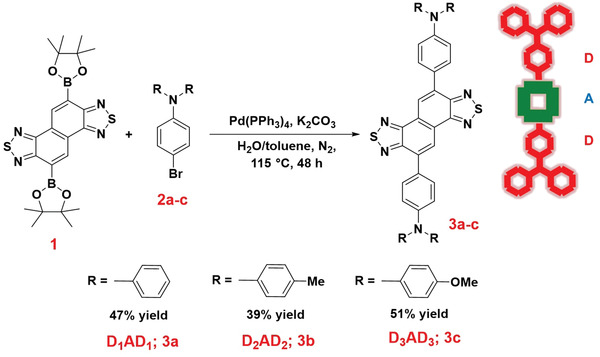
Reaction of different 4‐bromo‐*N*, *N*‐diarylaniline compounds with naphtho[1,2‐c:5,6‐c′bis[1,2,5]thiadiazole‐5,10‐diboronic acid bis(pinacol) ester. Reaction Conditions: Naphtho[1,2‐c:5,6‐c′bis[1,2,5]thiadiazole‐5,10‐diboronic acid bis(pinacol) ester, **1** (3.3 mmol), 4‐bromo‐*N*,*N*‐diarylaniline (8.2 mmol), Pd(PPh_3_)_4_ (0.17 mmol), K_2_CO_3_ (16.5 mmol), toluene (80 mL), H_2_O (10 mL), N_2_, 115 °C, 48 h.

We have synthesized three new D–A–D based small molecular NIR‐II dyes (**3a‐c**) where Naphtho[1,2‐c:5,6‐c′]bis[1,2,5]thiadiazole and N,N‐diarylaniline was used as a strong electron acceptor and donor respectively. These donor–acceptor–donor, D–A–D, based dyes were synthesized through carbon–carbon single bond formation via Suzuki cross‐coupling reaction.^[^ ^1^ ^]^ The scope of the synthetic procedure was employed for the reaction of a series of 4‐bromo‐*N*,*N*‐diarylaniline (**2a‐c**) with naphtho[1,2‐c:5,6‐c′]bis[1,2,5]thiadiazole‐5,10‐diboronic acid bis(pinacol) ester, **1**, at 115 °C for 48 h under nitrogen atmosphere (**Scheme** **1**). The reaction with bromo‐substituted *N*,*N*‐diphenylaniline, **2a**, produced **D_1_AD_1_**, **3a**, in 47% yield, while the bromoaniline having *N,N*‐ditolyl group, **2b**, afforded **D_2_AD_2_**, **3b**, in 39% yield. Furthermore, diboronic acid bis(pinacol) ester underwent reaction with bromoaniline containing *N,N*‐dimethoxy phenyl group, **2c**, to furnish the targeted product **D_3_AD_3_**, **3c**, in 51% yield. All the final products were characterized by mass spectrometry (Figures S1, S4, and S7, Supporting Information), ^1^H‐NMR (Figures S2, S5, and S8, Supporting Information), and ^13^C‐NMR (Figures S3, S6, and S9, Supporting Information) spectroscopy. The mass spectra represented the molecular ion peak (731.2014 for D_1_AD_1_; 825.3798 for D_2_AD_2_ and 854.5880 for D_3_AD_3_) for the synthesized compounds. NMR spectra showed the presence of only aromatic protons in case of D_1_AD_1_ compound. On the other hand, the presence of methyl protons (12 protons at 2.35 ppm) and methoxy protons (12 protons at 3.83 ppm) were visible in ^1^H‐NMR spectra for D_2_AD_2_ and D_3_AD_3_ compounds, respectively. The aliphatic carbon peaks were also visible in ^13^C‐NMR spectra for D_2_AD_2_ and D_3_AD_3_ compounds.

**Figure 1:**
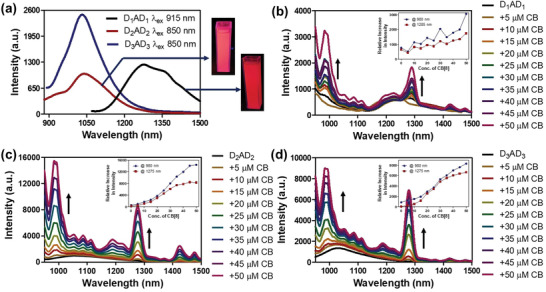
Emission spectra of a) D_1_AD_1_ upon excitation at 915 nm wavelength, D_2_AD_2_ and D_3_AD_3_ upon excitation at 850 nm wavelength. The color of the DMSO solution of the NIR‐II dyes has been showed in inset upon irradiation of UV light (365 nm). Emission spectra and (inset)emission intensity changes of b) D_1_AD_1_ at 915 nm, c) D_2_AD_2_ and d) D_3_AD_3_ at 850 nm upon gradual addition of CB[8] in DMSO solution.

Next, we studied UV–visible and fluorescence properties of the above‐synthesized dyes. The UV–vis spectrum of **D_1_AD_1_** showed a weak charge‐transfer absorption band between D–A–D unit at 915 nm in dimethyl sulfoxide (DMSO) solvent. The visible emission of the dye in DMSO showed an orange tint upon the irradiation of UV light (365 nm) (**Figure** **1**a). Interestingly, the solution of **D_1_AD_1_** when excited at 915 nm showed strong emission peak at 1281 nm with a small hump at 1356 nm (Figure 1a). This suggested the NIR‐II applicability of **D_1_AD_1_** dye. These observations were further confirmed by 2D excitation–emission studies (*λ*_ex_ 650–1000 nm; *λ*_em_ 1000–1400 nm) (Figure S10, Supporting Information). In contrast, **D_2_AD_2_** when substituted by the electron‐donating tetratolyl group showed a weak absorption peak at around 850 nm in DMSO solvent which upon excitation at the absorption maxima showed strong emission behavior at 1038 nm with strong blueshift emission of about 243 nm compared to **D_1_AD_1_** (Figure 1a). Similarly, **D_3_AD_3_** containing strong electron‐donating tetramethoxy phenyl group exhibited weak absorbance at around 850 nm, which upon excitation showed strong emission in NIR‐II region at 1031 nm with a small blueshift of about 7 nm compared to **D_2_AD_2_** in polar DMSO solvent (Figure 1a). A large stokes shift (≈366 nm) was observed for **D_1_AD_1_** which may indicate a fast relaxation from the initial state to the emissive state. This could also be due to intramolecular energy‐transfer between the donor and acceptor moieties.

Next, we studied the solubility behavior of these newly synthesized dyes in the presence of cucurbit[8]uril (**CB[8]**). CB[8] is widely used as a host molecule for several different guest molecules and is known to form supramolecular assembly due to the capture of those guest molecules into its hydrophobic cavity.^[^ ^59^ ^]^ We presumed that CB[8] could encapsulate neutral and electron‐donating groups present in the NIR‐II dyes in its nonpolar cavity and might form a unique supramolecular assembly. Accordingly, the NIR‐II dyes were mixed with CB[8] and their binding interactions were investigated by spectroscopic studies such as fluorescence and nuclear magnetic spectroscopy (NMR). The titration experiment was employed by the gradual addition of CB[8] in each NIR‐II dyes solution in DMSO. The overall fluorescence intensity increased significantly with a blueshift in emission upon the addition of an increasing amount of CB[8] to the solution of NIR‐II dyes. Moreover, a new peak with a redshift in emission was also generated. Thus, **D_1_AD_1_** showed a gradual increase in emission intensity at about 990 nm with the generation of two new peaks at about 1225 nm with weak blueshift emission of about 56 nm and at about 1290 nm with redshift emission of about 9 nm, respectively (Figure 1b). This result indicated the binding of **D_1_AD_1_** NIR‐II dye within the hydrophobic cavity of CB[8] which led to the improved solubilization of the dye and hence an increase in NIR‐II emission of the compounds. The fluorescence intensity of **D_2_AD_2_** also increased gradually with emission maxima at around 983–990 nm along with a blueshift of about 48 nm upon the addition of increasing concentrations of CB[8]. Furthermore, a new higher emission peak at around 1277–1280 nm emerged with a strong redshift emission of ≈242 nm (Figure 1c). Similarly, upon excitation at 850 nm, a gradual increase in fluorescence intensity was observed at about 990 nm with blueshift emission of 41 nm for **D_3_AD_3_** dye. Besides, one new peak with the hyperchromic emission at about 1280 nm and a strong redshift of ≈249 nm appeared when an increasing concentration of CB[8] was added to the solution of **D_3_AD_3_** NIR‐II dye (Figure 1d). The saturation in increment of emission intensity with a constant slope indicates the complete binding of the guest NIR‐II dyes with the host CB[8] molecules emphasizing the comprehensive formation of the host–guest complexes.

**Figure 2:**
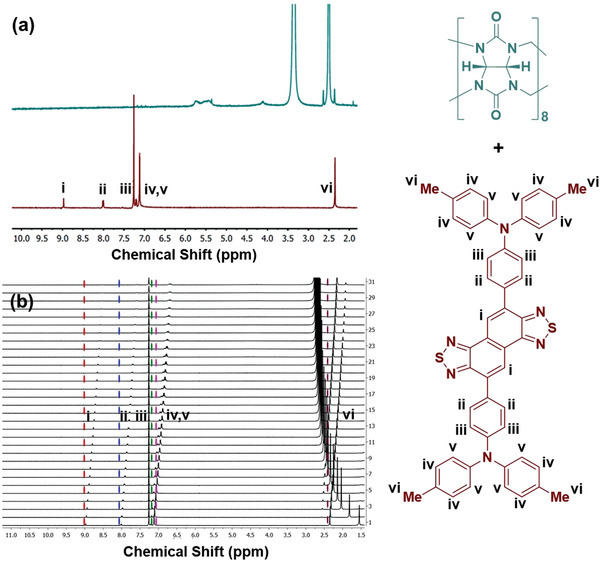
^1^H NMR spectra of a) CB[8] (green spectra) and D_2_AD_2_ (red spectra). The NMR experiments were carried out in d6‐DMSO solvent for CB[8] and CDCl_3_ solvent for D_2_AD_2_. b) NMR titration studies between D_2_AD_2_ and CB[8] demonstrate the formation of host–guest complex, D_2_AD_2_⊂CB[8]. The chemical shifts for 6 separate sets of protons (i–vi) are also assigned.

Furthermore, to confirm the binding of the NIR‐II dyes with CB[8], ^1^H‐NMR titration study was performed with one of our as‐synthesized **D_2_AD_2_** compound. A gradual up‐field chemical shift (*δ*) was observed when DMSO‐d_6_ solution of CB[8] was added in increasing concentration to the CDCl_3_ solution of **D_2_AD_2_** (**Figure** **2**). This shift may be attributed to the changes in the chemical environment of the dye protons that are located within the hydrophobic pocket of CB[8]. It is evident from the structure of **D_2_AD_2_** that out of six sets of chemically nonequivalent protons, napthobisthiadazole protons (*H*_i_) showed an upfield chemical shift (Δ*δ*) of ≈0.456 ppm during its titration with CB[8]. The chemical shifts of the proton (*H*_ii_ and *H*_iii_) residing on the aniline moiety showed an upfield shift of ≈0.427 and ≈0.47 ppm, respectively. On the other hand, the splitting pattern of the two chemically nonequivalent protons (*H*_iv_ and *H*_v_) of the phenyl group in *p*‐tolyl moiety of **D_2_AD_2_** appeared in multiplet. However, the splitting pattern of these protons changed completely during the titration and exhibited an upfield chemical shift of ≈0.432 ppm. Similarly, the resonance of the methyl proton (*H*_vi_) of *p*‐tolyl group was shifted ≈0.196 ppm upfield. This indicated an efficient host–guest complexation of **D_2_AD_2_** dye with CB[8].

**Figure 3:**
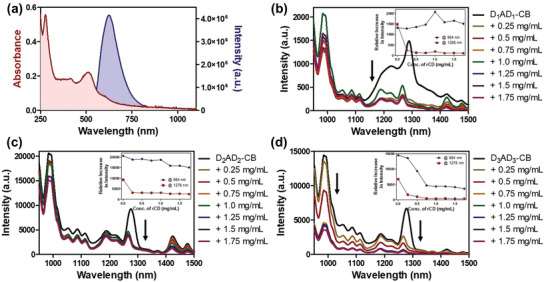
a) UV–vis and fluorescence spectra of red carbon dots. Emission spectra of b) D_1_AD_1_⊂CB, c) D_2_AD_2_⊂CB, and d) D_3_AD_3_⊂CB host–guest complexes upon gradual addition of aqueous solution of rCDots in DMSO solvent. D_1_AD_1_⊂CB was irradiated at 915 nm, whereas D_2_AD_2_⊂CB and D_3_AD_3_⊂CB complexes were irradiated at 850 nm.

Next, the user‐defined tunable photoluminescence properties of the formed supramolecular system were explored by the introduction of red‐emitting carbon dots (rCDots).^[^ ^59^ ^]^ The bright red luminescent CDots were prepared by hydrothermal method by mixing 2,5‐diaminotoluene sulfate and urea in an equivalent quantity (see the Supporting Information for more details).^[^ ^60^ ^]^ The as‐synthesized rCDots were then characterized by several physicochemical techniques such as UV–vis absorption, fluorescence, X‐ray photoelectron spectroscopy (XPS) and Fourier transform infrared (FTIR), transmission electron microscopy (TEM), and atomic force microscopy (AFM). UV–vis spectra showed the presence of two absorbance peaks at 276 and 510 nm (**Figure** **3**a). These peaks can be explained based on *π*–π* transition of aromatic C═C bonds and n–π* transition of C═N bonds present on the surface of the rCDots, respectively. Bright red fluorescence at 655 nm was also observed when the suspension was excited at 510 nm (Figure 3a). The morphological investigations of rCDots from TEM and AFM studies exhibited an average size of ≈10 nm by TEM and an average height of 2.5 ± 0.29 nm due to the flattening in anhydrous state (Figure S11, Supporting Information). Further FTIR and XPS measurements were carried out to confirm the functional groups, molecular structures or chemical bonds, and chemical states that were present on the surface of rCDots. The adventitious peak for C—C bond was located at 284.8 eV, while the peaks observed at 285.7 and 288.1 eV were attributed to C—O/C—N, and C═O/C═N, respectively. The decomposition of the N peak revealed the peaks at 398.8 and 400.1 and 404.1 eV which may be assigned to pyridinic, pyrrolic N, and nitrite nitrogen, respectively. For O1s region, two peaks were identified for C═O and CO/OH at 531.6 and 533.1 eV, respectively.

Finally, the investigation of the core state of S2p revealed a doublet at 168.1 and 169.3 eV related to C—SO*_x_* (*x* = 3, and 4) sulfurs (Figure S12, Supporting Information). On the other hand, the rCDot surface presented an abundance of polar functional groups with a strong band observed between 3100 and 3500 cm^−1^ which may be attributed to the stretching vibration of polar functional groups like O—H and N—H. The peak centered at 1131 cm^−1^ may be due to C—O. On the other hand, C═C which is typically from aromatic structures appeared between 1539 and 1635 cm^−1^. The other peak centered at 1448 cm^−1^ could be ascribed to C—N═ bond. Finally, the asymmetric stretching vibration of C—N and C—O could be detected at 1127–1159 cm^−1^ (Figure S13, Supporting Information).

The supramolecular coassemblies among rCDots and host–guest complexes of CB[8] and NIR‐II dyes were then investigated by fluorescence spectroscopy. A significant decrease in fluorescence intensity was observed when an increasing amount of rCDots was gradually added to the DMSO solution of the host–guest complexes (Figure 3). It was observed that both the fluorescence intensity peaks at 990 and 1225 nm decreased for **D_1_AD_1_** ⊂CB complex with the addition of increasing concentration of rCDots to the mixture once excited at 915 nm (Figure 3a). A similar trend was also noticed for the intensity peak at 1290 nm with a small blueshift emission of ≈23 nm. Furthermore, upon excitation at 850 nm, the fluorescence intensity of **D_2_AD_2_** ⊂CB complex decreased gradually along with a decrease in intensity peak at 1280 nm which was associated with a small hypsochromic shift of ≈15 nm (Figure 3b). Next, the decrease in emission intensity centered on around 990 and 1280 nm with a weak blueshift of ≈15 nm for **D_3_AD_3_** ⊂CB complex was also observed with the gradual addition of rCDots (Figure 3c). All of these results indicated the formation of coassembly among rCDots and the host–guest complexes formed between CB[8] and the newly synthesized NIR‐II dyes.

**Figure 4:**
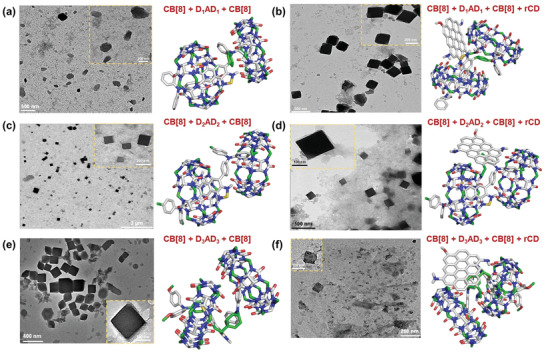
Transmission electron microscopic images of a) D_1_AD_1_⊂CB, c) D_2_AD_2_⊂CB, and e) D_3_AD_3_⊂CB host–guest complexes along with their most favorable docked geometries. Corresponding images of the supramolecular assemblies when red carbon dots are embedded in b) D_1_AD_1_⊂CB, d) D_2_AD_2_⊂CB, and f) D_3_AD_3_⊂CB complexes are also shown with their most favored docked geometries.

The morphologies of these supramolecular assemblies were then investigated under transmission electron microscope.^[^ ^61^ ^]^ It was revealed that supramolecular nanocubes with an average anhydrous diameter of ≈200 nm were formed when the NIR‐II dyes interacted with its host CB[8] molecules (**Figure** **4**a,c,e). Further, the structures of these fluorescent nanocubes remained almost intact with little distortion after their interaction with rCDots (Figure 4b,d,f) which quenched their fluorescence to a large extent. The observations were further corroborated from SEM images (Figure S14, Supporting Information).

We next sought further mechanistic insight for these structures using molecular docking and density functional theoretical calculations. First, we explored the stoichiometry between NIR‐II and CB[8] during the host–guest complexation and then the type of interaction between the rCDots and host–guest complexes. In this regard, the compounds were energy minimized by B3LYP/6‐31G(d) method in GAMESS software (Figure S15, Supporting Information). The highest occupied molecular orbital (HOMO)‐ lowest unoccupied molecular orbital (LUMO) levels were also calculated for the three NIR‐II dyes which indicated that HOMO is largely located over the donor moieties, whereas LUMO is located over the acceptor thiadiazole moieties (Figure S16, Supporting Information). The NIR‐II dyes and CB[8] were then docked in different stoichiometric ratios. The most favorable docked geometries for 1:1, 2:1, and 1:2 complexes between NIR‐II dyes and CB[8] are shown in Figure S17a–c,d–f (Supporting Information), and Figure 4, respectively.

**Figure 5:**
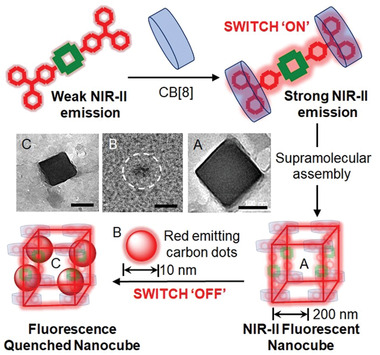
Schematic illustration of the switchable photoluminescence behavior via host–guest complexation between NIR‐II dyes and CB[8] followed by self‐aggregation with carbon dots. The scale bar of the inset TEM images indicates 100 nm for A and C and 5 nm for B.

It can be concluded from the comparative binding energies and conformational stability (i.e., minimal number of clustered geometries) of the host–guest complexes over the docked structures that 1:2 complexes between dye:CB[8] is the most energetically stable compared with other stoichiometries (Tables S1 and S2, Supporting Information). We also calculated HOMO and LUMO of the 1:2 complexes as reported in Figure S18 (Supporting Information). Next, to account for rCDots, a model was built based on ovalene.^[^ ^54^ ^]^ We monitored its interaction with the most favored geometries of 2:1 host–guest complexes in the next step (Figure 4; and Table S3, Supporting Information). It was observed that HOMO is largely located over ovalene (i.e., rCDots) whereas LUMO is located mostly over the host–guest complexes. The results suggested a plausible photoinduced transfer of electrons from rCDots to the NIR‐II dyes (Figure S19, Supporting Information) which can explain the observed quenching of photoluminescence of the host–guest complexes upon the addition of rCDots experimentally (**Figure** **5**). It can be concluded from the comparative binding energies and conformational stability (i.e., minimal number of clustered geometries) of the host–guest complexes over the docked structures that 1:2 complexes between dye:CB[8] are the most energetically stable compared with other stoichiometries (Tables S1 and S2, Supporting Information). This host–guest complexation between the dye and CB[8] molecules caused the observed changes in chemical environment of the dye protons (Figure 2b). The gradual up‐field chemical shift was therefore found for the D_2_AD_2_ protons whenever the inclusion complex formed in presence of CB[8] molecules. Our docking studies proved the phenomenon of complexation between the two molecules (Figure 4c). Furthermore, for better understanding of change in chemical environment of the dye protons, the docked structure of D_2_AD_2_ and CB[8] complex has been represented in two separate colors (Figure S20, Supporting Information). It was understood that the supramolecular assembly, discussed herein, can be better explained by single crystal structures. Although CB inclusion complexes were studied by single crystal geometries,^[^ ^62^, ^63^, ^64^ ^]^ carbon dots are generally amorphous in nature,^[^ ^65^, ^66^, ^67^, ^68^, ^69^, ^70^, ^71^, ^72^ ^]^ as observed in its powder X‐ray diffraction pattern, and do not crystallize. Hence, the formation of single crystals has not been attempted in this study.

UV–visible spectra of the DAD conjugates were shown in Figure S21 (Supporting Information). The concentration of D_1_AD_1_, D_2_AD_2_, and D_3_AD_3_ was set at 200 × 10^−6^ m. The DAD+CB conjugates were prepared by mixing 200 × 10^−6^ m of DAD and 400 × 10^−6^ m of CB[8] solutions in DMSO. Further, DAD+CB+rCD conjugates were made with 200 × 10^−6^ m of DAD, 400 × 10^−6^ m of CB[8] solutions in DMSO and 1 mg mL^−1^ solution of rCD in water. The DAD compounds represented quite broad absorption at higher wavelength and the DAD+CB conjugates showed remarkably enhanced absorbance. The numerical sum value of DAD and CB absorbances is significantly lower than the absorbance of DAD+CB conjugate. The enhanced absorbance of the conjugate clearly indicated that the DAD and CB mixture is not merely a physical mixture, but a host–guest complexation among the two.^[^ ^73^, ^74^ ^]^ Moreover, the absorption spectra got redshifted upon the addition of rCDots to the DAD+CB conjugate. This bathochromic shift can be attributed to the change in functional groups on the surface of the conjugate.^[^ ^75^ ^]^ In addition, we have measured the quantum yields (QYs) of the three DAD systems before and after the addition of CB and rCD to validate the observed phenomenon of switchable photoluminescence. It was found that the quantum yield of the DAD systems increased after the addition of CB[8], however it decreased nearly to the original value after the addition of rCDots to the dye and CB[8] conjugate (**Table** **1**). It was also observed that the quantum yield of D_3_AD_3_ and CB conjugate was the highest among others. Representative integrated fluorescence intensity plots for D_3_AD_3_ systems in comparison to the reference near‐infrared fluorophore, IR‐26, were shown in Figure S22 (Supporting Information).

**Table 1:** Quantum yield measurements of the three DAD systems before and after the addition of CB and rCD. The values were calculated with respect to a reference near‐infrared fluorophore, IR‐26 (QY = 0.5%)

Compounds	Quantum yield [%]
D_1_AD_1_	0.121
D_1_AD_1_ + CB	0.203
D_1_AD_1_ + CB + CD	0.124
D_2_AD_2_	0.157
D_2_AD_2_ + CB	0.261
D_2_AD_2_ + CB + CD	0.179
D_3_AD_3_	0.317
D_3_AD_3_ + CB	0.524
D_3_AD_3_ + CB + CD	0.338

**Figure 6:**
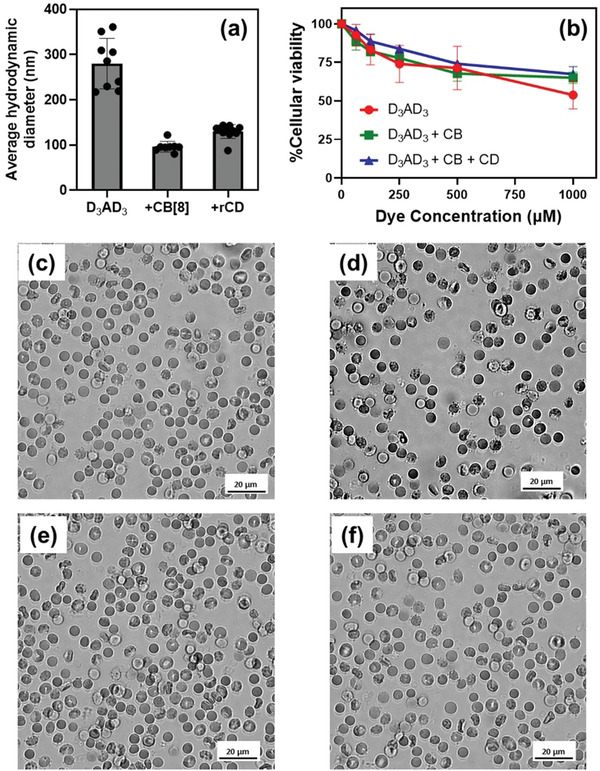
a) Average hydrodynamic diameter of D_3_AD_3_ NIR‐II dye before and after the addition of CB[8] and rCD demonstrating the dispersibility of the particles in water. b) Cellular toxicity of D_3_AD_3_ conjugates before and after the addition of CB[8] and rCD in HEK293 cells. Blood biocompatibility of d) 400 × 10^−6^ m of D_3_AD_3_, e) 400 × 10^−6^ m of D_3_AD_3_ and 800 × 10^−6^ m of CB[8], and f) 400 × 10^−6^ m of D_3_AD_3_, 800 × 10^−6^ m of CB[8] and 2 mg mL^−1^ solution of rCD in 1X PBS compared to c) nontreated blood.

Inspired by the superior quantum yield of the inclusion complex (D_3_AD_3_+CB[8]), comparable to the commercially available near‐infrared fluorophore, IR‐26, we thought of engaging ourprobe for bioimaging studies.^[^ ^75^ ^]^ First, we investigated the dispersibility of our probe in water. Light scattering studies demonstrated that the dye suspension showed the formation of large aggregates with limited suspend‐ability in water. However, the supramolecular host–guest complex (photoluminescence “ON”) and its conjugate with rCDots (photoluminescence “OFF”) demonstrated the formation of smaller sized particles with improved stability in water (**Figure** **6**a). Indeed, the purpose of introducing CB[8], carbon dots and their complexation with the NIR‐II probes was twofold, i.e., to impart improved aqueous dispersibility and to introduce a switch on and off properties in these probes.

**Figure 7:**
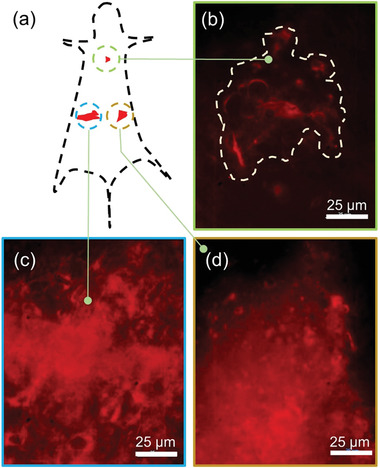
Ex vivo NIR‐II imaging for tissue and surgical navigation: a) Site of the agent administration; b) heart; c) colon and d) under the skin tissue recorded within 60 min after the contrast injection. Scale bar: 25 µm.

The biocompatibility of these aggregates was first investigated in HEK293 cells by 3‐(4,5‐dimethylthiazol‐2‐yl)‐2,5‐diphenyl‐2H‐tetrazolium bromide (MTT) based cell vibility assay. The aggregates formed with D_3_AD_3_ NIR‐II dye (Figure 6b) was found to be fairly nontoxic compared to the D_1_AD_1_ and D_2_AD_2_ dye conjugates (Figure S23, Supporting Information). The turn‐ON photoluminescent complex, D_3_AD_3_+CB[8], was found to be quite biocompatible in nature even at higher concentrations. This observation may be attributed to the superior dispersibility of D_3_AD_3_+CB[8] conjugate in water. The blood compatibility of D_3_AD_3_ NIR‐II dye conjugates was then monitored with insignificant change in blood morphology (Figure 6c–f). These in vitro results inspired us to investigate the ex vivo NIR‐II bioimaging^[^ ^76^, ^77^, ^78^, ^79^, ^80^, ^81^ ^]^ capabilities of the turn‐ON photoluminescent complex, D_3_AD_3_+CB[8], for tissue and surgical navigation. The site of agent administration is shown in **Figure** **7**a. The fluorescent images of heart (Figure 7b), colon (Figure 7c), and under the skin tissue (Figure 7d) were then visualized under the NIR‐II camera within 60 min after the contrast injection. The regions were found to be NIR‐II visible with good fluorescence contrast demonstrating improved in vivo penetration depth of this inclusion complex (D_3_AD_3_+CB[8]).

## 3 Conclusion

In conclusion, three small molecular NIR‐II emissive probes were reported herein which upon sequential addition of cucurbit[8]uril and red‐emitting CDots demonstrated tunable changes in photoluminescence behavior. All the dyes exhibited appreciable Stokes shifts which is principally important for practical applications of fluorescence because it allows separating (strong) excitation light from (weak) emitted fluorescence when using appropriate optics. Nanocube‐like morphologies were observed for both the supramolecular assemblies formed before and after the addition of rCDots to the host–guest complexes of CB[8] and NIR‐II dyes. The phenomenon was investigated by ^1^H‐NMR, molecular docking, and theoretical density functional theory (DFT) calculations to explain the potential assembly mechanism. At least one of the developed turn‐ON inclusion complex showed improved quantum yield, aqueous dispersibility, cellular viability, blood compatibility, and NIR‐II bioimaging capability. Although the advantages of NIR‐II probes are well documented, the organic probes reported suffered from major drawbacks, i.e., i) poor aqueous solubility hampering their bioimaging capabilities, ii) inadequate QY for effective in vivo imaging for sparse biological markers and iii) lack of flexibility to tune the fluorescence properties. The current work addressed all these critical issues to offer a combination of organic and nanoparticle‐based self‐assembled probe with improved QY, aqueous dispersibility and optical properties, that is tunable to “switch on and off” fluorescence. To the best of our knowledge, this is one of the first report of NIR‐II switchable fluorescence driven by host–guest complexation and supramolecular self‐assembly. With these promising results, we warrant in‐depth preclinical evaluation of these agents in our laboratory.

## Conflict of Interest

The authors declare no conflict of interest.

## Supporting information

Supporting Information

## Data Availability

The data that support the findings of this study are available from the corresponding author upon reasonable request.

## References

[1] Q. T. Nguyen , R. Y. Tsien , Nat. Rev. Cancer 2013, 13, 653. 23924645 10.1038/nrc3566 PMC4427343

[2] S. Keereweer , P. B. Van Driel , T. J. Snoeks , J. D. Kerrebijn , R. J. B. de Jong , A. L. Vahrmeijer , H. J. Sterenborg , C. W. Löwik , Clin. Cancer Res. 2013, 19, 3745. 23674494 10.1158/1078-0432.CCR-12-3598

[3] D. Pan , M. Pramanik , S. A. Wickline , L. v. Wang , G. M. Lanza , Contrast Media Mol. Imaging 2011, 6, 378. 22025338 10.1002/cmmi.449

[4] P. Mukherjee , S. K. Misra , M. C. Gryka , H.‐H. Chang , S. Tiwari , W. L. Wilson , J. W. Scott , R. Bhargava , D. Pan , Small 2015, 11, 4691. 25994248 10.1002/smll.201500728

[5] C. T. N. Pham , D. G. Thomas , J. Beiser , L. M. Mitchell , J. L. Huang , A. Senpan , G. Hu , M. Gordon , N. A. Baker , D. Pan , G. M. Lanza , D. E. Hourcade , Nanomedicine 2014, 10, 651. 24211337 10.1016/j.nano.2013.10.012 PMC3966962

[6] F. Ding , Y. Zhan , X. Lu , Y. Sun , Chem. Sci. 2018, 9, 4370. 29896378 10.1039/c8sc01153b PMC5961444

[7] D. Pan , M. Pramanik , A. Senpan , S. A. Wickline , L. v. Wang , G. M. Lanza , J. Nanosci. Nanotechnol. 2010, 10, 8118. 21121304 10.1166/jnn.2010.3034 PMC3096062

[8] M. H. Ross , A. K. Esser , G. C. Fox , A. H. Schmieder , X. Yang , G. Hu , D. Pan , X. Su , Y. Xu , D. v. Novack , T. Walsh , G. A. Colditz , G. H. Lukaszewicz , E. Cordell , J. Novack , J. A. J. Fitzpatrick , D. L. Waning , K. S. Mohammad , T. A. Guise , G. M. Lanza , K. N. Weilbaecher , Cancer Res. 2017, 77, 6299. 28855208 10.1158/0008-5472.CAN-17-1225 PMC5841166

[9] R. Tian , Q. Zeng , S. Zhu , J. Lau , S. Chandra , R. Ertsey , K. S. Hettie , T. Teraphongphom , Z. Hu , G. Niu , Sci. Adv. 2019, 5, eaaw0672. 31548981 10.1126/sciadv.aaw0672 PMC6744268

[10] M. S. Khan , S. K. Misra , K. Dighe , Z. Wang , A. S. Schwartz‐Duval , D. Sar , D. Pan , Biosens. Bioelectron. 2018, 110, 132. 29605712 10.1016/j.bios.2018.03.044

[11] D. Pan , A. H. Schmieder , K. Wang , X. Yang , A. Senpan , G. Cui , K. Killgore , B. Kim , J. S. Allen , H. Zhang , S. D. Caruthers , B. Shen , S. A. Wickline , G. M. Lanza , Theranostics 2014, 4, 565. 24723979 10.7150/thno.7581 PMC3982128

[12] S. Zhu , Z. Hu , R. Tian , B. C. Yung , Q. Yang , S. Zhao , D. O. Kiesewetter , G. Niu , H. Sun , A. L. Antaris , X. Chen , Adv. Mater. 2018, 30, 1802546. 10.1002/adma.201802546 29985542

[13] I. Srivastava , S. K. Misra , F. Ostadhossein , E. Daza , J. Singh , D. Pan , Nano Res. 2017, 10, 3269.

[14] D. Pan , Mol. Pharmaceutics 2013, 10, 781. 10.1021/mp400044j 23452025

[15] S. K. Misra , H.‐H. Chang , P. Mukherjee , S. Tiwari , A. Ohoka , D. Pan , Sci. Rep. 2015, 5, 14986. 26462751 10.1038/srep14986 PMC4604511

[16] S. He , J. Song , J. Qu , Z. Cheng , Chem. Soc. Rev. 2018, 47, 4258. 29725670 10.1039/c8cs00234g

[17] M. S. Khan , K. Dighe , Z. Wang , I. Srivastava , E. Daza , A. S. Schwartz‐Dual , J. Ghannam , S. K. Misra , D. Pan , Analyst 2018, 143, 1094. 29387841 10.1039/c7an01932g

[18] S. K. Misra , F. Ostadhossein , E. Daza , E. v. Johnson , D. Pan , Adv. Funct. Mater. 2016, 26, 8031.

[19] Y. Liu , Y. Yang , M. Sun , M. Cui , Y. Fu , Y. Lin , Z. Li , L. Nie , Chem. Sci. 2017, 8, 2710. 28451353 10.1039/c6sc04798j PMC5399633

[20] Q. Yu , S. Huang , Z. Wu , J. Zheng , X. Chen , L. Nie , J. Nucl. Med. 2020, 61, 1079. 31806769 10.2967/jnumed.119.233155 PMC7383080

[21] Y. Liu , H. Liu , H. Yan , Y. Liu , J. Zhang , W. Shan , P. Lai , H. Li , L. Ren , Z. Li , L. Nie , Adv. Sci. 2019, 6, 1801615. 10.1002/advs.201801615 PMC6469237 31016108

[22] Y. Fan , P. Wang , Y. Lu , R. Wang , L. Zhou , X. Zheng , X. Li , J. A. Piper , F. Zhang , Nat. Nanotechnol. 2018, 13, 941. 30082923 10.1038/s41565-018-0221-0

[23] K. Shou , C. Qu , Y. Sun , H. Chen , S. Chen , L. Zhang , H. Xu , X. Hong , A. Yu , Z. Cheng , Adv. Funct. Mater. 2017, 27, 1700995. 29623009 10.1002/adfm.201700995 PMC5879786

[24] G. Hong , A. L. Antaris , H. Dai , Nat. Biomed. Eng. 2017, 1, 0010.

[25] K. K. Liu , S. Y. Song , L. Z. Sui , S. X. Wu , P. T. Jing , R. Q. Wang , Q. Y. Li , G. R. Wu , Z. Z. Zhang , K. J. Yuan , C. X. Shan , Adv. Sci. 2019, 6, 1900766. 10.1002/advs.201900766 PMC6724478 31508282

[26] Z. Ma , M. Zhang , J. Yue , C. Alcazar , Y. Zhong , T. C. Doyle , H. Dai , N. F. Huang , Adv. Funct. Mater. 2018, 28, 1803417. 31327961 10.1002/adfm.201803417 PMC6640151

[27] X. D. Zhang , H. Wang , A. L. Antaris , L. Li , S. Diao , R. Ma , A. Nguyen , G. Hong , Z. Ma , J. Wang , Adv. Mater. 2016, 28, 6872. 27253071 10.1002/adma.201600706 PMC5293734

[28] Z. Feng , X. Yu , M. Jiang , L. Zhu , Y. Zhang , W. Yang , W. Xi , G. Li , J. Qian , Theranostics 2019, 9, 5706. 31534513 10.7150/thno.31332 PMC6735390

[29] J. Qi , C. Sun , A. Zebibula , H. Zhang , R. T. Kwok , X. Zhao , W. Xi , J. W. Lam , J. Qian , B. Z. Tang , Adv. Mater. 2018, 30, 1706856. 10.1002/adma.201706856 29341330

[30] L. Li , Z. Yang , S. Zhu , L. He , W. Fan , W. Tang , J. Zou , Z. Shen , M. Zhang , L. Tang , Adv. Mater. 2019, 31, 1901187. 10.1002/adma.201901187 30957918

[31] H. Wan , H. Ma , S. Zhu , F. Wang , Y. Tian , R. Ma , Q. Yang , Z. Hu , T. Zhu , W. Wang , Adv. Funct. Mater. 2018, 28, 1804956. 31832053 10.1002/adfm.201804956 PMC6907024

[32] B. Guo , Z. Feng , D. Hu , S. Xu , E. Middha , Y. Pan , C. Liu , H. Zheng , J. Qian , Z. Sheng , Adv. Mater. 2019, 31, 1902504. 10.1002/adma.201902504 31169334

[33] A. Zebibula , N. Alifu , L. Xia , C. Sun , X. Yu , D. Xue , L. Liu , G. Li , J. Qian , Adv. Funct. Mater. 2018, 28, 1703451.

[34] M. Zhang , J. Yue , R. Cui , Z. Ma , H. Wan , F. Wang , S. Zhu , Y. Zhou , Y. Kuang , Y. Zhong , Proc. Natl. Acad. Sci. USA 2018, 115, 6590. 29891702 10.1073/pnas.1806153115 PMC6042152

[35] P. Moitra , M. Alafeef , K. Dighe , M. Frieman , D. Pan , ACS Nano 2020, 14, 7617. 32437124 10.1021/acsnano.0c03822

[36] K. Welsher , Z. Liu , S. P. Sherlock , J. T. Robinson , Z. Chen , D. Daranciang , H. Dai , Nat. Nanotechol. 2009, 4, 773. 10.1038/nnano.2009.294 PMC2834239 19893526

[37] K. Welsher , S. P. Sherlock , H. Dai , Proc. Natl. Acad. Sci. USA 2011, 108, 8943. 21576494 10.1073/pnas.1014501108 PMC3107273

[38] G. Hong , J. C. Lee , J. T. Robinson , U. Raaz , L. Xie , N. F. Huang , J. P. Cooke , H. Dai , Nat. Med. 2012, 18, 1841. 23160236 10.1038/nm.2995 PMC3595196

[39] Y. Dai , D. Yang , D. Yu , C. Cao , Q. Wang , S. Xie , L. Shen , W. Feng , F. Li , ACS Appl. Mater. Interfaces 2017, 9, 26674. 28726368 10.1021/acsami.7b06109

[40] Y. Zhong , Z. Ma , S. Zhu , J. Yue , M. Zhang , A. L. Antaris , J. Yuan , R. Cui , H. Wan , Y. Zhou , Nat. Commun. 2017, 8, 737. 28963467 10.1038/s41467-017-00917-6 PMC5622117

[41] D. Rosenblum , N. Joshi , W. Tao , J. M. Karp , D. Peer , Nat. Commun. 2018, 9, 1410. 29650952 10.1038/s41467-018-03705-y PMC5897557

[42] W. Wang , Z. Ma , S. Zhu , H. Wan , J. Yue , H. Ma , R. Ma , Q. Yang , Z. Wang , Q. Li , Y. Qian , C. Yue , Y. Wang , L. Fan , Y. Zhong , Y. Zhou , H. Gao , J. Ruan , Z. Hu , Y. Liang , H. Dai , Adv. Mater. 2018, 30, 1800106. 10.1002/adma.201800106 PMC6485425 29682821

[43] A. L. Antaris , H. Chen , S. Diao , Z. Ma , Z. Zhang , S. Zhu , J. Wang , A. X. Lozano , Q. Fan , L. Chew , Nat. Commun. 2017, 8, 15269. 28524850 10.1038/ncomms15269 PMC5454457

[44] Z. Sheng , B. Guo , D. Hu , S. Xu , W. Wu , W. H. Liew , K. Yao , J. Jiang , C. Liu , H. Zheng , Adv. Mater. 2018, 30, 1800766. 10.1002/adma.201800766 29806179

[45] a) Q. Zhang , Q. Guo , Q. Chen , X. Zhao , S. J. Pennycook , H. Chen , Adv. Sci. 2020, 7, 1902576; 10.1002/advs.201902576 PMC7141019 32274298

[46] M. Alafeef , P. Moitra , D. Pan , Biosens. Bioelectron. 2020, 165, 112276. 32729465 10.1016/j.bios.2020.112276

[47] Y. Wang , X. H. Liu , Q. Wang , M. Quick , M. A. Kovalenko , Q. Y. Chen , N. Koch , N. Pinna , Angew. Chem., Int. Ed. 2020, 59, 7748. 10.1002/anie.201915074 PMC7317755 32068941

[48] B. Shan , T. T. Li , M. K. Brennaman , A. Nayak , L. Wu , T. J. Meyer , J. Am. Chem. Soc. 2019, 141, 463. 30525576 10.1021/jacs.8b11110

[49] S. Dong , B. Zheng , F. Wang , F. Huang , Acc. Chem. Res. 2014, 47, 1982. 24684594 10.1021/ar5000456

[50] J. Liu , Y. Lan , Z. Yu , C. S. Tan , R. M. Parker , C. Abell , O. A. Scherman , Acc. Chem. Res. 2017, 50, 208. 28075551 10.1021/acs.accounts.6b00429 PMC5474693

[51] F. Ostadhossein , S. K. Misra , P. Mukherjee , A. Ostadhossein , E. Daza , S. Tiwari , S. Mittal , M. C. Gryka , R. Bhargava , D. Pan , Small 2016, 12, 5845. 27545321 10.1002/smll.201601161 PMC5542878

[52] X. L. Ni , S. Chen , Y. Yang , Z. Tao , J. Am. Chem. Soc. 2016, 138, 6177. 27123563 10.1021/jacs.6b01223

[53] Q. W. Zhang , D. Li , X. Li , P. B. White , J. Mecinovic , X. Ma , H. Agren , R. J. Nolte , H. Tian , J. Am. Chem. Soc. 2016, 138, 13541. 27652689 10.1021/jacs.6b04776

[54] I. Srivastava , J. S. Khamo , S. Pandit , P. Fathi , X. Huang , A. Cao , R. T. Haasch , S. Nie , K. Zhang , D. Pan , Adv. Funct. Mater. 2019, 29, 1902466.

[55] F. Ostadhossein , L. Benig , I. Tripathi , S. K. Misra , D. Pan , ACS Appl. Mater. Interfaces 2018, 10, 19408. 29757601 10.1021/acsami.8b03727

[56] F. Ostadhossein , D. Pan , Wiley Interdiscip. Rev.: Nanomed. Nanobiotechnol. 2017, 9, e1436. 10.1002/wnan.1436 27791335

[57] P. Fathi , J. S. Khamo , X. Huang , I. Srivastava , M. B. Esch , K. Zhang , D. Pan , Carbon 2019, 145, 572. 10.1016/j.carbon.2018.12.105 PMC8596966 34795455

[58] H. A. Nguyen , I. Srivastava , D. Pan , M. Gruebele , ACS Nano 2020, 14, 6127. 32324372 10.1021/acsnano.0c01924

[59] H. Wu , Y. Chen , X. Dai , P. Li , F. Stoddart , Y. Liu , J. Am. Chem. Soc. 2019, 141, 6583. 30860369 10.1021/jacs.8b13675

[60] F. Ostadhossein , D. Sar , I. Tripathi , J. Soares , E. E. Remsen , D. Pan , ACS Appl. Mater. Interfaces 2020, 12, 10183. 32031773 10.1021/acsami.0c00705

[61] X. Wu , Y. Chen , Q. Yu , F. Q. Li , Y. A. Liu , Chem. Commun. 2019, 55, 4343. 10.1039/c9cc01601e 30911744

[62] P. Wang , Y. Wu , Y. Zhao , Y. Yu , M. Zhang , L. Cao , Chem. Commun. 2017, 53, 5503. 10.1039/c7cc02074k 28418058

[63] S. Combes , K. T. Tran , M. M. Ayhan , H. Karoui , A. Rockenbauer , A. Tonetto , V. Monnier , L. Charles , R. Rosas , S. Viel , D. Siri , P. Tordo , S. Clair , R. Wang , D. Bardelang , O. Ouari , J. Am. Chem. Soc. 2019, 141, 5897. 30808163 10.1021/jacs.9b00150

[64] J. del Barrio , J. Liu , R. A. Brady , C. S. Y. Tan , S. Chiodini , M. Ricci , R. Fernández‐Leiro , C.‐J. Tsai , P. Vasileiadi , L. di Michele , D. Lairez , C. Toprakcioglu , O. A. Scherman , J. Am. Chem. Soc. 2019, 141, 14021. 31422657 10.1021/jacs.9b07506 PMC6772898

[65] I. Srivastava , P. Moitra , D. Sar , K. Wang , M. Alafeef , J. Scott , D. Pan , Nanoscale 2021, 13, 16288. 34558578 10.1039/d1nr02786g

[66] I. Srivastava , P. Moitra , M. Fayyaz , S. Pandit , T. L. Kampert , P. Fathi , H. R. Alanagh , K. Dighe , M. Alafeef , K. Vuong , M. Jabeen , S. Nie , J. Irudayaraj , D. Pan , ACS Appl. Mater. Interfaces 2021, 13, 59747. 34878252 10.1021/acsami.1c19995

[67] P. Fathi , P. Moitra , M. M. McDonald , M. B. Esch , D. Pan , Nanoscale 2021, 13, 13487. 34477753 10.1039/d1nr01295a

[68] I. Srivastava , J. S. Khamo , S. Pandit , P. Fathi , X. Huang , A. Cao , R. T. Haasch , S. Nie , K. Zhang , D. Pan , Adv. Funct. Mater. 2019, 29, 1902466.

[69] P. Ray , P. Moitra , D. Pan , VIEW 2022, 3, 20200089.

[70] P. Fathi , A. Roslend , K. Mehta , P. Moitra , K. Zhang , D. Pan , Nanoscale 2021, 13, 4785. 33434263 10.1039/d0nr08485a PMC9297654

[71] M. Alafeef , K. Dighe , P. Moitra , D. Pan , ACS Sustainable Chem. Eng. 2022, 10, 245. 35036178 10.1021/acssuschemeng.1c06066

[72] F. Ostadhossein , P. Moitra , E. Altun , D. Dutta , D. Sar , I. Tripathi , S.‐H. Hsiao , V. Kravchuk , S. Nie , D. Pan , Commun. Biol. 2021, 4, 846. 34267305 10.1038/s42003-021-02372-y PMC8282845

[73] Y. Zhang , S. Zhang , Z. Zhang , L. Ji , J. Zhang , Q. Wang , T. Guo , S. Ni , R. Cai , X. Mu , W. Long , H. Wang , Front. Chem. 2021, 9, 728066. 34395388 10.3389/fchem.2021.728066 PMC8358119

[74] B. Geng , W. Shen , P. Li , F. Fang , H. Qin , X. K. Li , D. Pan , L. Shen , ACS Appl. Mater. Interfaces 2019, 11, 44949. 31714729 10.1021/acsami.9b15569

[75] C. Shen , Q. Lou , J. Zang , K. Liu , S. Qu , L. Dong , C. Shan , Adv. Sci. 2020, 7, 1903525. 10.1002/advs.201903525 PMC7175254 32328432

[76] P. Moitra , M. Alafeef , K. Dighe , Z. Sheffield , D. Dahal , D. Pan , Chem. Commun. 2021, 57, 6229. 10.1039/d1cc01410b 34048518

[77] M. Alafeef , P. Moitra , K. Dighe , D. Pan , Nat. Protoc. 2021, 16, 3141. 33931780 10.1038/s41596-021-00546-w

[78] H. Ma , C. Liu , Z. Hu , P. Yu , X. Zhu , R. Ma , Z. Sun , C.‐H. Zhang , H. Sun , S. Zhu , Y. Liang , Chem. Mater. 2020, 32, 2061.

[79] R. Tian , Q. Zeng , S. Zhu , J. Lau , S. Chandra , R. Ertsey , K. S. Hettie , T. Teraphongphom , Z. Hu , G. Niu , D. O. Kiesewetter , H. Sun , X. Zhang , A. L. Antaris , B. R. Brooks , X. Chen , Sci. Adv. 2019, 5, eaaw0672. 31548981 10.1126/sciadv.aaw0672 PMC6744268

[80] S. Zhu , B. C. Yung , S. Chandra , G. Niu , A. L. Antaris , X. Chen , Theranostics 2018, 8, 4141. 30128042 10.7150/thno.27995 PMC6096392

[81] A. L. Antaris , H. Chen , S. Diao , Z. Ma , Z. Zhang , S. Zhu , J. Wang , A. X. Lozano , Q. Fan , L. Chew , M. Zhu , K. Cheng , X. Hong , H. Dai , Z. Cheng , Nat. Commun. 2017, 8, 15269. 28524850 10.1038/ncomms15269 PMC5454457

