# Supplementary material for: Small Molecule NIR‐II Dyes for Switchable Photoluminescence via Host –Guest Complexation and Supramolecular Assembly with Carbon Dots
Source: Adv Sci (Weinh). 2022 Jun 3;9(22):2202414. doi: 10.1002/advs.202202414 (PMC9353451; doi:10.1002/advs.202202414)
Supplement: Supplementary file 1 — Supporting Information [file ADVS-9-2202414-s001.pdf]

ADVANCED SCIENCE
Open Access

# Supporting Information

for *Adv. Sci.*, DOI 10.1002/advs.202202414

Small Molecule NIR-II Dyes for Switchable Photoluminescence via Host –Guest Complexation and Supramolecular Assembly with Carbon Dots

*Dinabandhu Sar, Fatemeh Ostadhossein, Parikshit Moitra, Maha Alafeef and Dipanjan Pan**

# Supporting Information

**Small Molecule NIR-II Dyes for Switchable Photoluminescence via Host -Guest Complexation and Supramolecular Assembly with Carbon Dots**

*Dinabandhu Sar,[†] Fatemeh Ostadhossein,[†] Parikshit Moitra,[†] Maha Alafeef and Dipanjan Pan**

Corresponding author: dipanjan@som.umaryland.edu

## 1. Experimental Section.

**Materials and Methods.**

All the reagents and chemicals were purchased from well-known commercial vendors and used without further purification steps. Cucurbit[8]uril was procured from Sigma-Aldrich and milli-Q water was used throughout the experiments.

**Synthetic Procedure of NIR-II Dyes.**

Naphtho[1,2-c:5,6-c′]bis[1,2,5]thiadiazole-5,10-diboronic acid bis(pinacol) ester, **1** (3.3 mmol), tetrakis(triphenylphosphine)palladium(0) (0.17 mmol), potassium carbonate (16.5 mmol) and 4-bromo-*N*,*N*-diarylaniline, **2** (8.2 mmol) were mixed and dissolved in anhydrous toluene (80 mL) and nitrogen-bubbled distilled water (10 mL). Either of 4-Bromotriphenylamine, **2a**, 4-Bromo-N,N-di-p-tolylaniline, **2b** or 4-Bromo-N,N-bis(4-methoxyphenyl)aniline, **2c**, was used as the bromo components to synthesize three different compounds. This solution was heated at 115 °C for 48 hours under nitrogen atmosphere. After the reaction time is over, the solvent was removed under reduced pressure and the resulting solid was dissolved in chloroform. This organic layer was washed with brine and filtered through anhydrous sodium sulphate. The filtrate was dried in a rotary evaporator and the crude product was purified by silica gel column chromatography with hexane/$CHCl_3$ as the eluent. This afforded the final compounds: 4,4'-(naphtho[1,2-c:5,6-c']bis([1,2,5]thiadiazole)-5,10-

diyl)bis(N,N-diphenylaniline), $D_1AD_1$, **3a** in 47% yield; 4,4'-(naphtho[1,2-c:5,6-c']bis([1,2,5]thiadiazole)-5,10-diyl)bis(N,N-di-p-tolylaniline), $D_2AD_2$, **3b** in 39% yield; 4,4'-(naphtho[1,2-c:5,6-c']bis([1,2,5]thiadiazole)-5,10-diyl)bis(N,N-bis(4-methoxyphenyl)aniline), $D_3AD_3$, **3c** in 51% yield.

**Characterization of 3a.**

$^1$H NMR (400 MHz, $CDCl_3$, TMS, rt) δ (ppm) 9.01 (*m*, 2H), 8.91 (*d*, J = 8.0 Hz, 2H), 8.10 (*m*, 2H), 8.03 (*m*, 4H), 7.30 (*m*, 8H), 7.21 (*m*, 8H), 7.08 (*m*, 4H); $^{13}$C NMR (100 MHz, $CDCl_3$, TMS, rt) δ (ppm) 147.58, 130.48, 129.66, 127.66, 125.29, 124.48, 123.76, 122.90, 120.78. HRMS (m/z, ESI-MS, $[M+H]^+$) Calcd. for $C_{46}H_{31}N_6S_2$ 731.2052, found 731.2014.

**Characterization of 3b.**

$^1$H NMR (400 MHz, $CDCl_3$, TMS, rt) δ (ppm) 8.97 (*s*, 2H), 8.0 (*d*, J = 8.0 Hz, 4H), 7.19 (*d*, J = 8.0 Hz, 4H), 7.12 (*m*, 16H), 2.35 (*s*, 12H); $^{13}$C NMR (100 MHz, $CDCl_3$, TMS, rt) δ (ppm) 153.01, 147.84, 143.85, 132.18, 132.0, 129.0, 124.22, 123.22, 120.5, 30.41, 29.16, 28.68, 21.67, 19.86, 13.10. HRMS (m/z, ESI-MS, $[M+K]^+$) Calcd. for $C_{50}H_{38}KN_6S_2$ 825.2236, found 825.3798.

**Characterization of 3c.**

$^1$H NMR (400 MHz, $CDCl_3$, TMS, rt) δ (ppm) 8.95 (*s*, 2H), 7.98 (*d*, J = 8.0 Hz, 4H), 7.16 (*d*, J = 8.0 Hz, 8H), 7.10 (*d*, J = 8.0 Hz, 4H), 6.88 (*m*, 8H), 3.83 (*s*, 12H); $^{13}$C NMR (100 MHz, $CDCl_3$, TMS, rt) δ (ppm) 156.26, 154.04, 149.33, 140.46, 132.98, 130.03, 128.47, 127.16, 127.11, 124.96, 124.01, 119.68, 114.81, 55.52, 31.93, 30.19, 29.71, 29.37, 22.70, 14.19. HRMS (m/z, MALDI, $[M+4H]^+$) Calcd. for $C_{50}H_{42}N_6O_4S_2$ 854.2709, found 854.5880.

**Synthesis of Red Carbon Dot.**

The synthesis of red carbon dot was carried out following a previously reported literature procedure.[1] Briefly, equimolar amount of 2,5-diaminotoluene sulfate (100 M) and urea (100 M) were dissolved in 10 mL of milli-Q water and vortexed for 2 mins. The well suspended solution was then transferred to a teflon-lined autoclave for hydrothermal synthesis when the heating cycle was set to 150 °C for 8 h. After the required incubation time at elevated temperature, the vessel was brought down to room temperature, added with milli-Q water and centrifuged for 10 mins at 4200 rpm to remove the undesired contaminants. The nanoparticles were passed through two consecutive syringe filters (0.45 and 0.22 μm mesh size) and then dialyzed (MWCO = 2000 Da) against 1X PBS buffer (pH = 7.4). The suspension was kept at 4 °C for long term storage and tip sonicated briefly before each experiment.

**UV-Visible Spectroscopy.**

The UV-Visible spectra were recorded on Agilent Cary 5000 UV-Vis-NIR system.

**Fluorescence Spectroscopy.**

The fluorescence spectra were recorded on a Varian Cary 5G instrument. The 2D excitation–emission contours were obtained from a Horiba Aqualog scanning spectrofluorometer (Horiba Scientific, Edison, NJ).

**NMR Spectroscopy.**

The NMR spectra were collected on Varian VXR 500 (Varian, Inc., Palo Alto, CA) spectrometer operating at 400 MHz. The results were analyzed by MestRenova 11.0 (Mestrelab Research SL; Santiago de Compostela, Spain) software.

**Mass Spectrometry.**

Both the electrospray ionization (ESI) and matrix-assisted laser desorption/ionization (MALDI) quadrapole time of flight (Q-ToF) mass spectra were collected at mass spec facilities at UIUC.

**FT-IR Spectroscopy.**

The samples were drop casted and dried on MirrIR IR-reflective glass slides (Kevley Technologies, Chesterland, OH, USA). The thin films were then recorded in attenuated total reflectance (ATR) mode on a Nicolet Nexus 670 FT-IR instrument (ThermoFisher Scientific, Waltham, MA, US).

**Scanning Electron Microscopy (SEM).**

The samples were mounted on a SEM sample holder using a piece of carbon tape and then gold-coated via sputtering for 60 sec at 20 mA. The imaging was performed with an accelerating voltage of 10 kV, extracting current of 10 µA and in a relatively long working distance of 25 mm. These SEM images were recorded with a Hitachi (Schaumburg, Illinois) S-4800 SEM with Oxford Instruments (Abingdon, Oxfordshire).

**Transmission Electron Microscopy (TEM).**

The TEM images were obtained using a TEM microscope (FEI) equipped with a Peltiercooled Tietz (TVIPS) 2k × 2k charge-coupled device camera. 5 µL of the diluted samples were deposited on a carbon-coated copper grid (TED PELLA Inc., Redding, CA) and kept for 5 minutes. The extra liquid was then removed with filter paper and the sample was dried overnight before imaging.

**Atomic Force Microscopy (AFM).**

The atomic force microscopy was performed in tapping mode with a Bruker MultiMode Nanoscope IIIA instrument (Billerica, MA). The diluted samples were deposited on a mica wafer fixed on a steel disc and the extra liquid was taken out after 5 mins. The disc was then vacuum dried for overnight.

**X-ray Photoelectron Spectroscopy (XPS).**

The carbon dots were drop cast over a glass slide and dried properly before the XPS measurement on a Physical Electronics PHI 5400 spectrometer using Al Kα (1486.6 eV) radiation. The spectra were corrected to the C–C bond at 284.8 eV and the analyses were performed on CasaXPS software.[2]

**Docking Studies.**

The chemical structures were first energy minimized using a general ab initio quantum chemistry package, General Atomic and Molecular Electronic Structure System (GAMESS) program.[3] We used B3LYP functional while performing the density functional theoretical (DFT) calculations with 6-31G(d) as the basis set. Pople N31 was used for the polar groups. The energy minimized structures were then undertaken for docking studies using AutoDock 4.0 software.[4]

**HOMO-LUMO Calculations.**

The chemical structures were initially energy optimized and the HOMO-LUMO surfaces were then calculated from their energy minimized geometries using GAMESS.[3] We used B3LYP functional while performing the density functional theoretical (DFT) calculations with 6-31G(d) as the basis set. Pople N31 was used for the polar groups. The highest occupied molecular orbital (HOMO) and the lowest unoccupied molecular orbital (LUMO) was then

calculated from the energy minimized geometries. The HOMO and LUMO were also calculated for the docked geometries in a similar fashion.

**Quantum yield measurements.**

The quantum yields of the conjugates were measured according to the previously reported methods.[5–7] UV-visible and fluorescence spectra of the DAD systems before and after the addition of CB and rCD were collected at five different concentrations. The maximum concentration of DAD systems was 200 µM. The DAD+CB conjugates were prepared by mixing 200 µM of DAD and 400 µM of CB[8] solutions in DMSO. Further, DAD+CB+rCD conjugates were made with 200 µM of DAD, 400 µM of CB[8] solutions in DMSO and 1 mg/mL solution of rCD in water. These solutions were then diluted sequentially to obtain five different concentrations for quantum yield measurements.

Integrated fluorescence intensities were calculated and plotted against the absorbances recorded at the excitation wavelength for five different concentrations. The curves were linear fitted and the slopes of the curves were used to calculated quantum yield of the respective systems. The following equation was used to calculate the quantum yields.

$$QY_X = QY_{ST} \times \left(\frac{Slope_X}{Slope_{ST}}\right) \times \left(\frac{RI_X^2}{RI_{ST}^2}\right)$$

Here, $QY_X$ and $QY_{ST}$ are the quantum yields for the sample and standard, IR-26 dye respectively. $RI_X$ and $RI_{ST}$ are the refractive indexes for the solvents used for suspending sample and standard respectively. A reference near-infrared fluorophore, IR-26 (QY = 0.5%), was used to calculate the quantum yields.

**Measurement of hydrodynamic diameter.**

A DMSO stock solution of the NIR-II dye (1 mM) was first prepared and mixed with a DMSO stock solution of CB[8] (2 mM) to form the inclusion complex (photoluminescence ‘ON’). This

supramolecular host-guest complex was further incubated with a 5 mg/mL water solution of rCD (photoluminescence 'OFF'). This solution was then diluted at least 100 times with milli-Q water before any measurement. The average hydrodynamic diameter of these particles was then monitored with a particle tracking analyzer (Zetaview Particle Metrix). An average of 10 such measurements was represented in each case. The machine cell was adequately cleaned before each use.

**MTT based cellular viability assay.**

HEK293 cells were cultured in DMEM high glucose media with 10% FBS supplement. Cells were maintained and passaged at required intervals with 0.25% trypsin-EDTA media. Cytotoxicity of the three NIR-II dye conjugates (before and after the addition of CB[8] and rCD) at different concentrations was determined using MTT assay. A stock solution of NIR-II dye (10 mM), CB[8] (20 mM) and rCD (50 mg/mL) was used for preparing the suspensions of definite concentrations.

In brief, 20000 cells per well were plated in 96 well plate and allowed to grow till 70-80% confluency. After 24 h, the cells were treated with different concentrations of the conjugates for 6 h. After the incubation period, the media was removed, and the cells were added with fresh media. After another 42 h of incubation, 20 µL of MTT (5 mg/mL stock concentration) was added to the wells for 4 h. The media was finally removed and added with 200 µL of DMSO. The % cellular viability was then calculated as,

% Cellular viability = [{($A_{570}$ (treated cells) – $A_{570}$ (DMSO)] / [($A_{570}$ (non-treated cells) – $A_{570}$ (DMSO)} × 100]

**Blood smear experiment.**

The protocol was followed from a previously reported literature protocol.[8] Human blood was incubated with 400 µM of $D_3AD_3$, 800 µM of CB[8] and 2 mg/mL solution of rCD in 1X PBS.

The blood samples were then diluted 5 times, allowed to air-dry for 1-2 mins over a glass slide added with a coverslip before being imaged under bright field Nikon Ti2 inverted microscope (100x).

**NIR-II imaging studies.**

A BALB/c mouse was sacrificed and injected with the turn-ON photoluminescent complex, $D_3AD_3$+CB[8], for *ex vivo* NIR-II bioimaging. The inclusion complex was made with 400 µM of $D_3AD_3$ and 800 µM of CB[8]. 20 µL of this suspension were then injected either to heart or colon or under the skin tissue and incubated for 30 mins at room temperature. The tissues were then imaged immediately under Nikon Ti2-E Inverted Automated Research Microscope. For NIR-II imaging, NIRvana 640ST camera was used. ET850/40x Chroma ET Series excitation filter and ET1300/100m Emission Filter 25mm with custom 50/50 beam splitter was used for NIR-II microscopic imaging.

## 2. Figures.

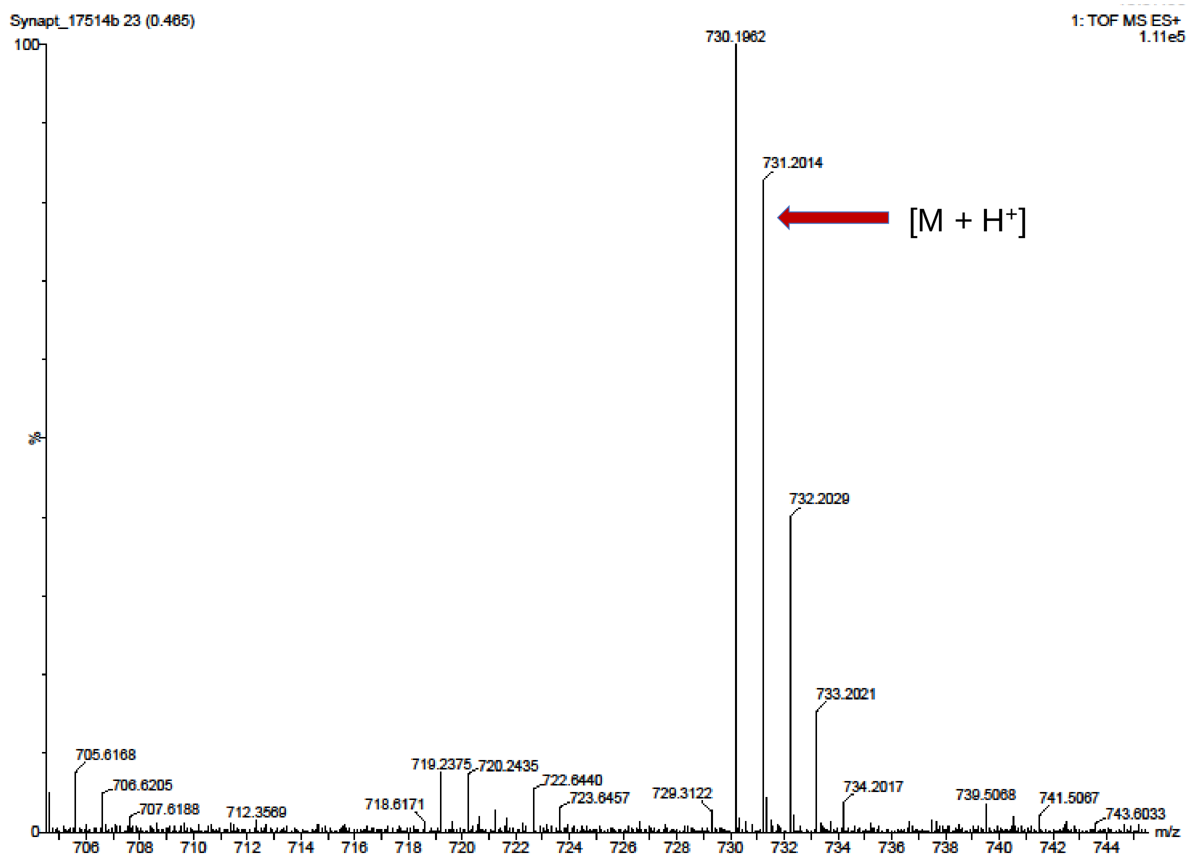


**Figure S1.** HRMS of $D_1AD_1$ compound.

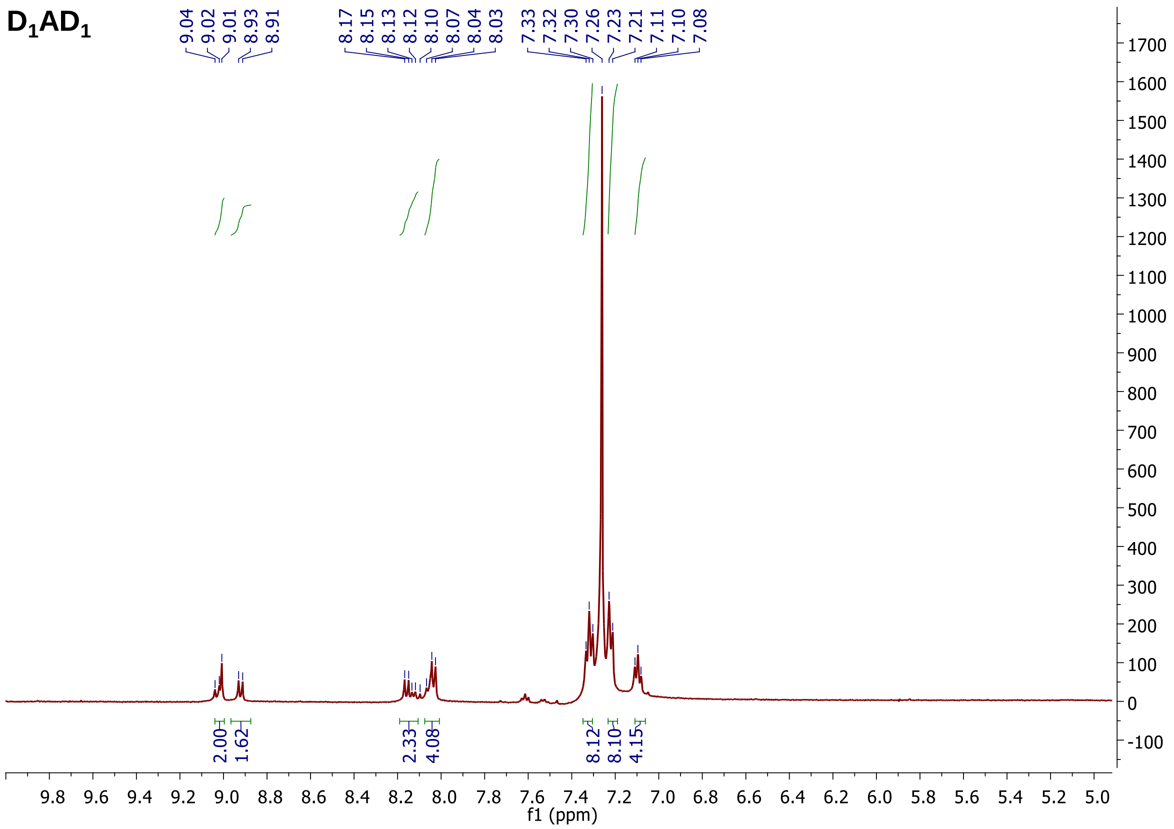


**Figure S2.** $^{1}$H-NMR spectrum of $D_1AD_1$ compound.

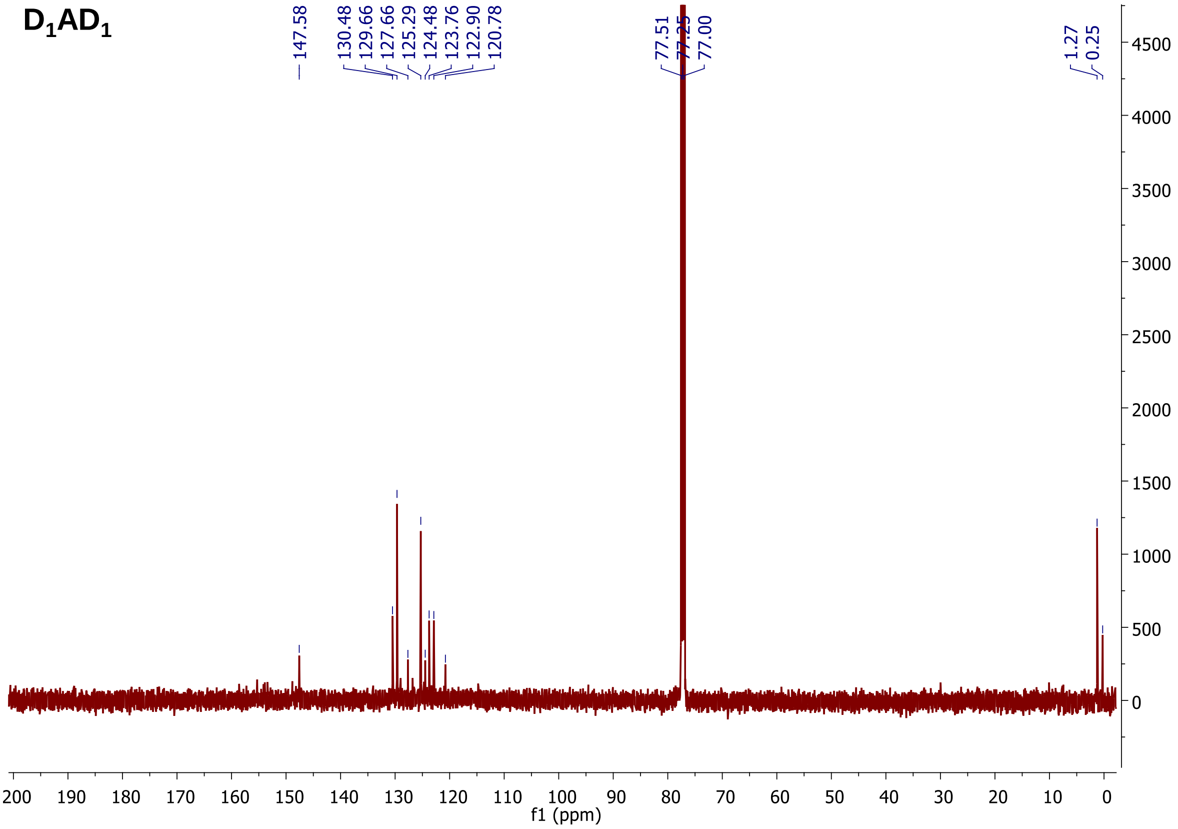


**Figure S3.** $^{13}$C-NMR spectrum of $D_1AD_1$ compound.

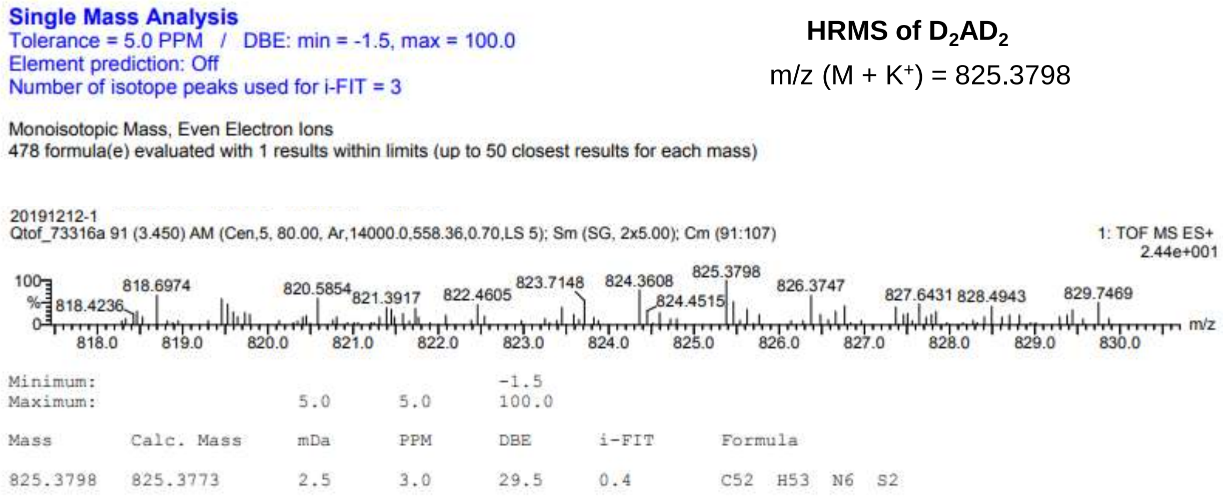


**Figure S4.** HRMS of $D_2AD_2$ compound.

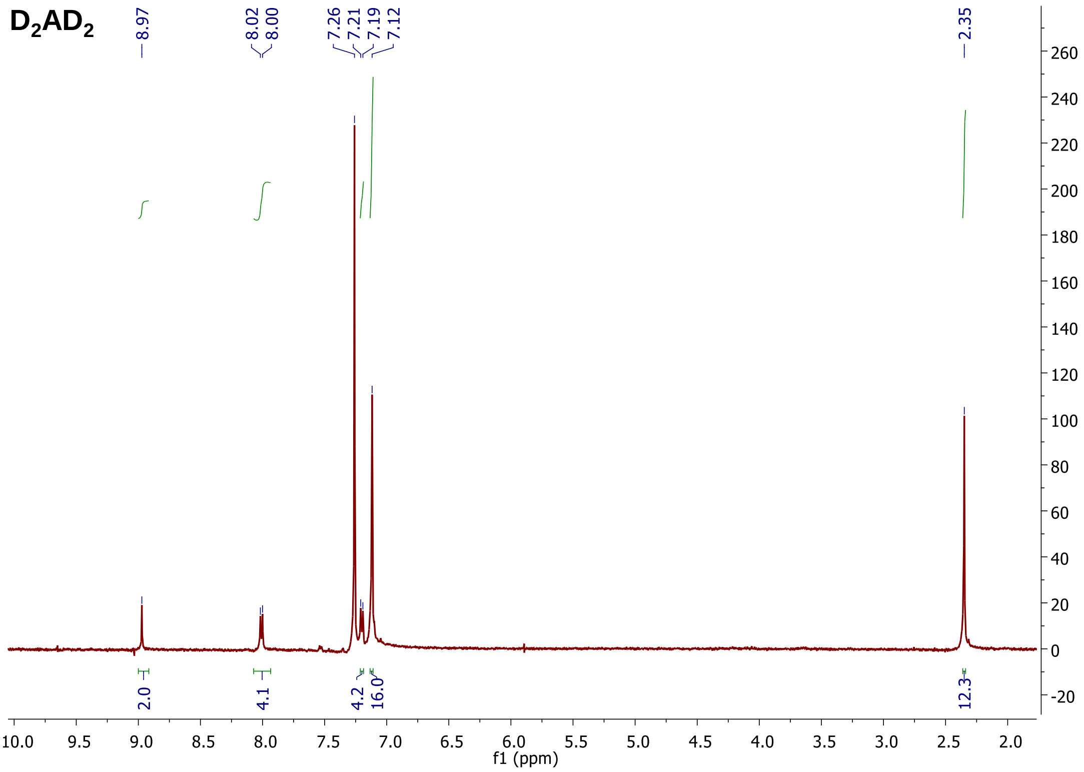


**Figure S5.** $^{1}$H-NMR spectrum of $D_2AD_2$ compound.

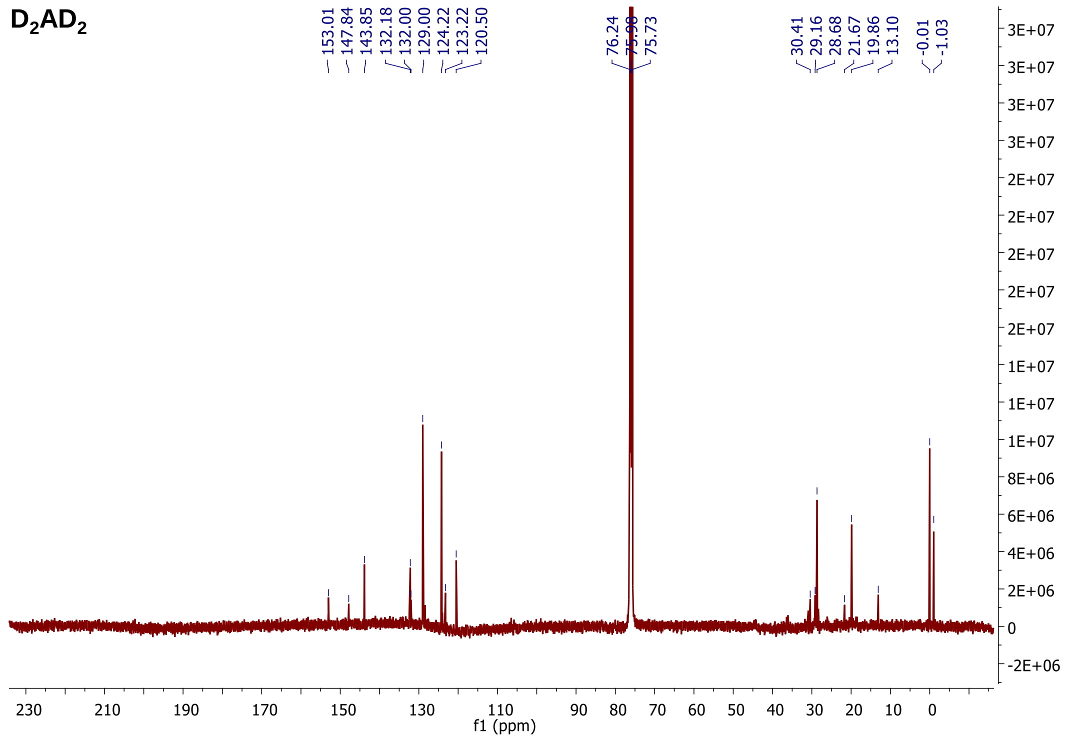


**Figure S6.** $^{13}$C-NMR spectrum of $D_2AD_2$ compound.

**Single Mass Analysis**
Tolerance = 5.0 PPM / DBE: min = -1.5, max = 100.0
Element prediction: Off
Number of isotope peaks used for i-FIT = 3

Monoisotopic Mass, Even Electron Ions
4659 formula(e) evaluated with 20 results within limits (up to 50 closest results for each mass)

**HRMS of $D_3AD_3$**
m/z (M + 4H$^+$)= 854.5880

DS-130-N
Qtof_73351 64 (2.437) AM (Cen,5, 80.00, Ar,14000.0,716.46,0.70,LS 5); Sm (SG, 2x5.00); Cm (57:77-10:21x8.000)
1: TOF MS ES+
2.60e+001

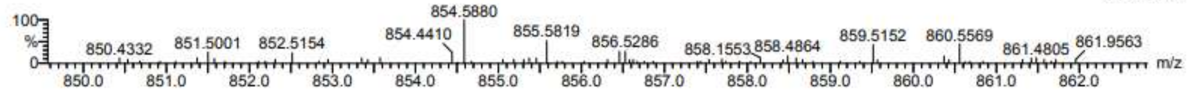


| | | | | | | |
|---|---|---|---|---|---|---|
| Minimum: | | | | -1.5 | | |
| Maximum: | | 5.0 | 5.0 | 100.0 | | |
| Mass | Calc. Mass | mDa | PPM | DBE | i-FIT | Formula |
| 854.5880 | 854.5879 | 0.1 | 0.1 | 7.5 | 3.1 | C48 H85 N3 O4 Na S2 |
| | 854.5883 | -0.3 | -0.4 | 20.5 | 2.7 | C54 H76 N7 S |
| | 854.5876 | 0.4 | 0.5 | 24.5 | 2.4 | C61 H76 N O2 |
| | 854.5884 | -0.4 | -0.5 | 13.5 | 1.0 | C48 H77 N7 O5 Na |
| | 854.5886 | -0.6 | -0.7 | 16.5 | 2.8 | C56 H81 N O2 Na S |
| | 854.5870 | 1.0 | 1.2 | 15.5 | 2.5 | C53 H80 N3 O4 S |
| | 854.5893 | -1.3 | -1.5 | 12.5 | 3.2 | C49 H81 N7 Na S2 |
| | 854.5863 | 1.7 | 2.0 | 6.5 | 2.9 | C45 H84 N5 O6 S2 |
| | 854.5859 | 2.1 | 2.5 | 17.5 | 2.4 | C52 H77 N7 Na S |
| | 854.5903 | -2.3 | -2.7 | 10.5 | 3.3 | C50 H84 N3 O4 S2 |
| | 854.5908 | -2.8 | -3.3 | 16.5 | 1.2 | C50 H76 N7 O5 |
| | 854.5852 | 2.8 | 3.3 | 21.5 | 2.1 | C59 H77 N O2 Na |
| | 854.5910 | -3.0 | -3.5 | 19.5 | 3.1 | C58 H80 N O2 S |
| | 854.5911 | -3.1 | -3.6 | 12.5 | 1.3 | C52 H81 N O7 Na |
| | 854.5849 | 3.1 | 3.6 | 25.5 | 2.0 | C57 H72 N7 |
| | 854.5846 | 3.4 | 4.0 | 12.5 | 2.2 | C51 H81 N3 O4 Na S |
| | 854.5917 | -3.7 | -4.3 | 15.5 | 3.4 | C51 H80 N7 S2 |
| | 854.5918 | -3.8 | -4.4 | 8.5 | 1.7 | C45 H81 N7 O5 Na S |
| | 854.5919 | -3.9 | -4.6 | 11.5 | 3.6 | C53 H85 N O2 Na S2 |
| | 854.5839 | 4.1 | 4.8 | 3.5 | 2.7 | C43 H85 N5 O6 Na S2 |

**Figure S7.** HRMS of $D_3AD_3$ compound.

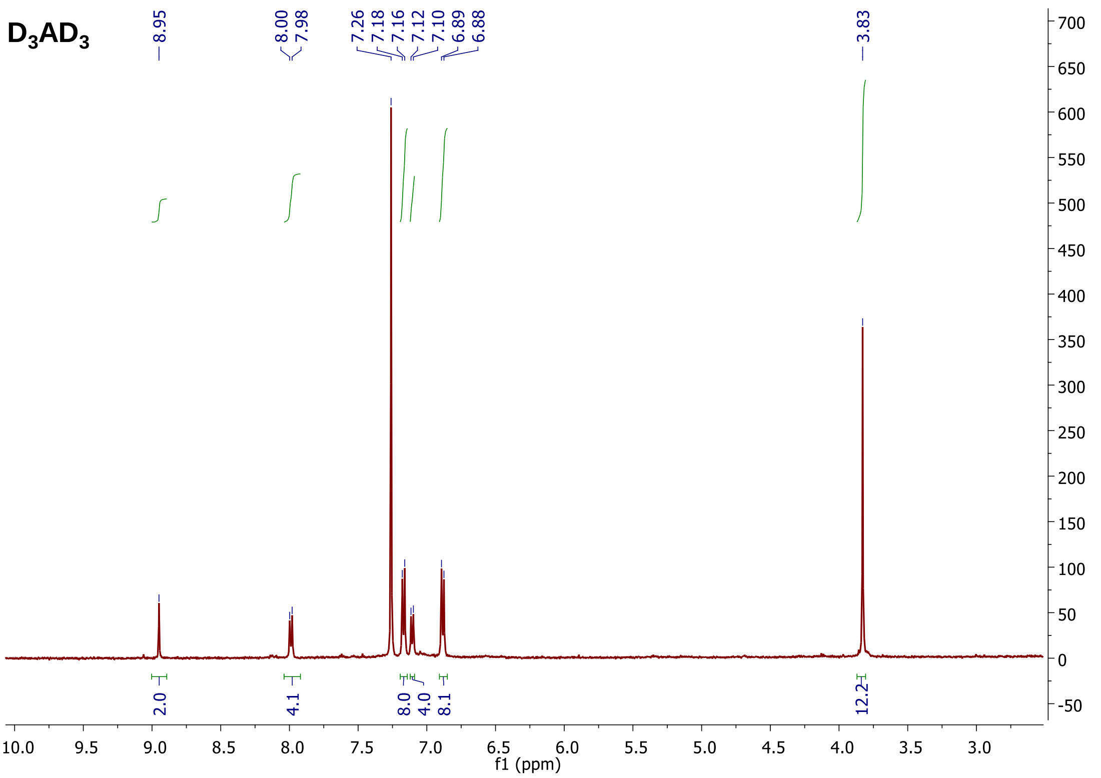


**Figure S8.** $^1$H-NMR spectrum of $D_3AD_3$ compound.

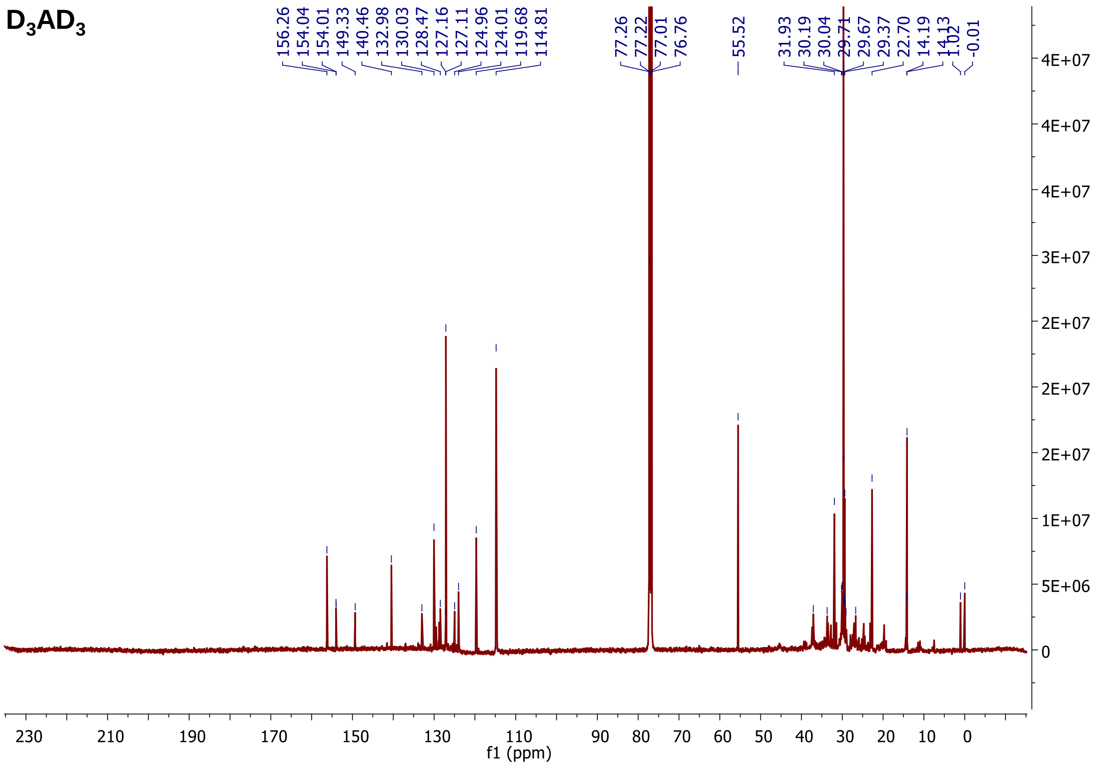


**Figure S9.** $^{13}$C-NMR spectrum of $D_3AD_3$ compound.

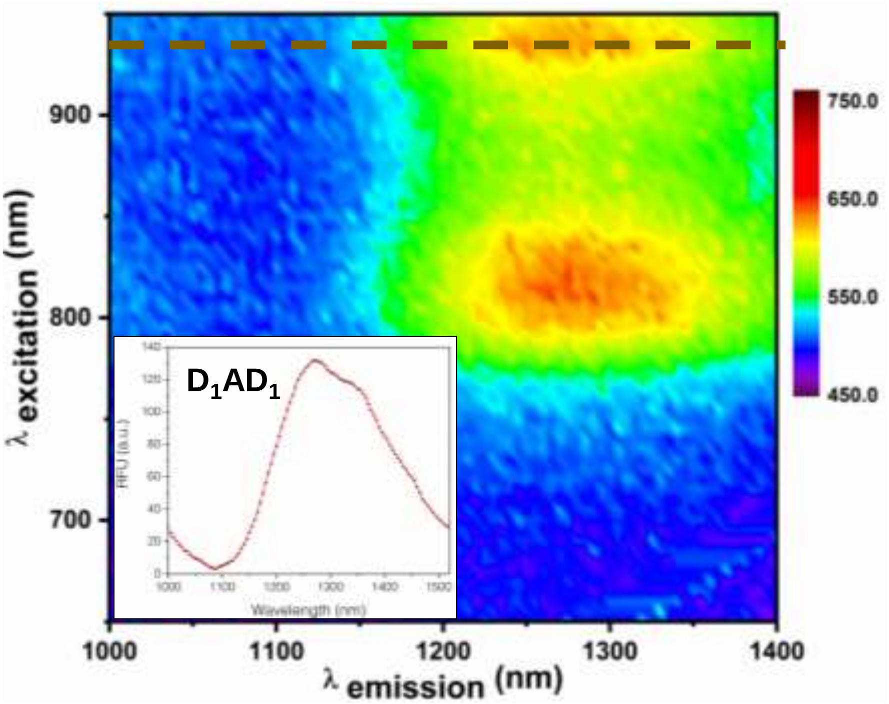


**Figure S10.** 2D excitation emission contour profile of $D_1AD_1$ dye.

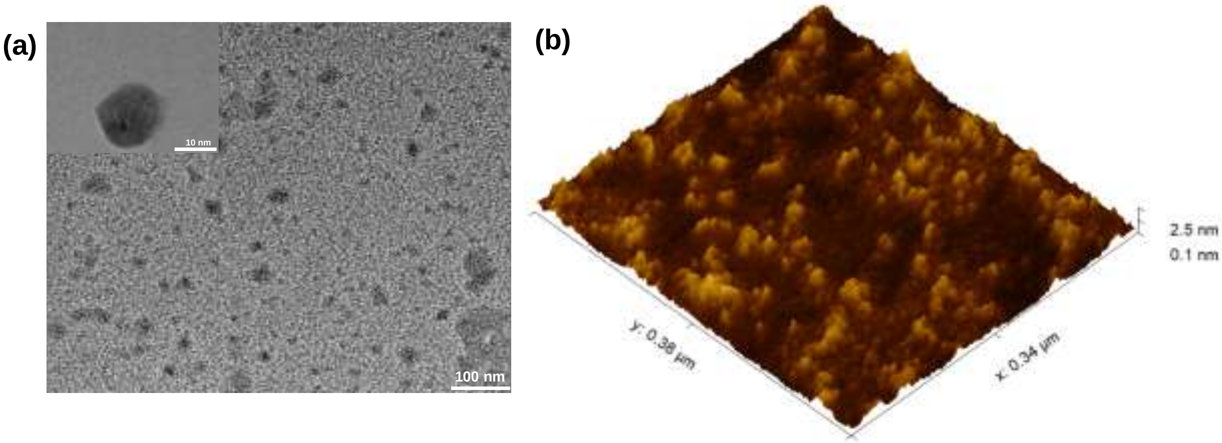


**Figure S11.** (a) Transmission electron microscopic and (b) atomic force microscopic images of the synthesized red carbon dots.

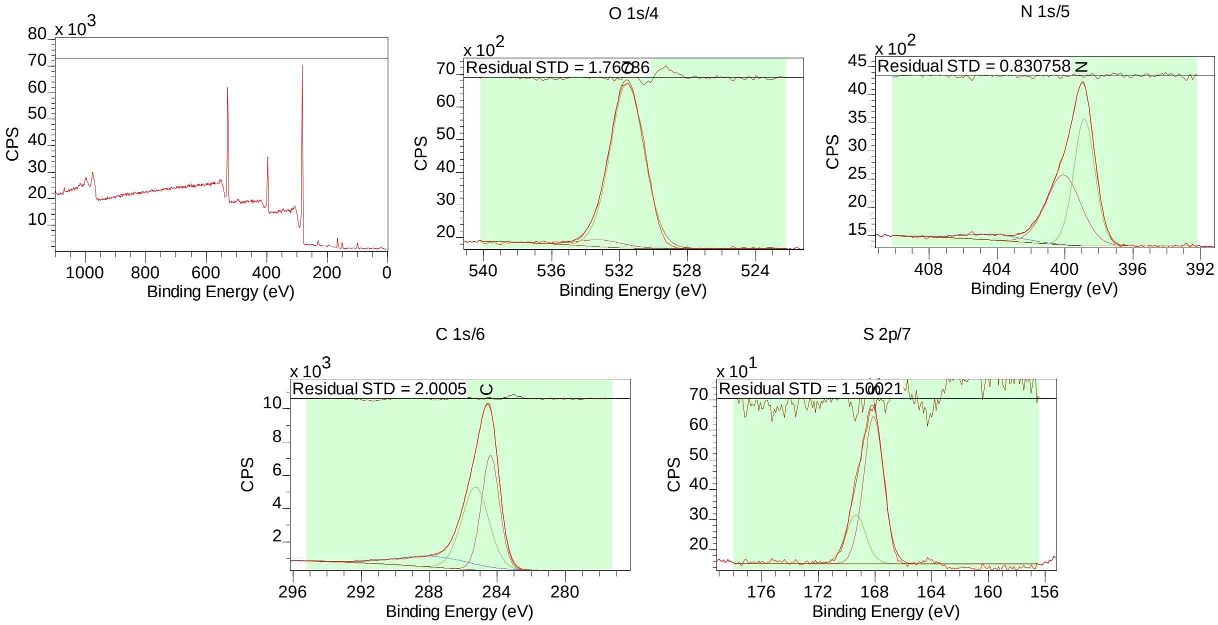


**Figure S12.** XPS profile for the synthesized red carbon dots.

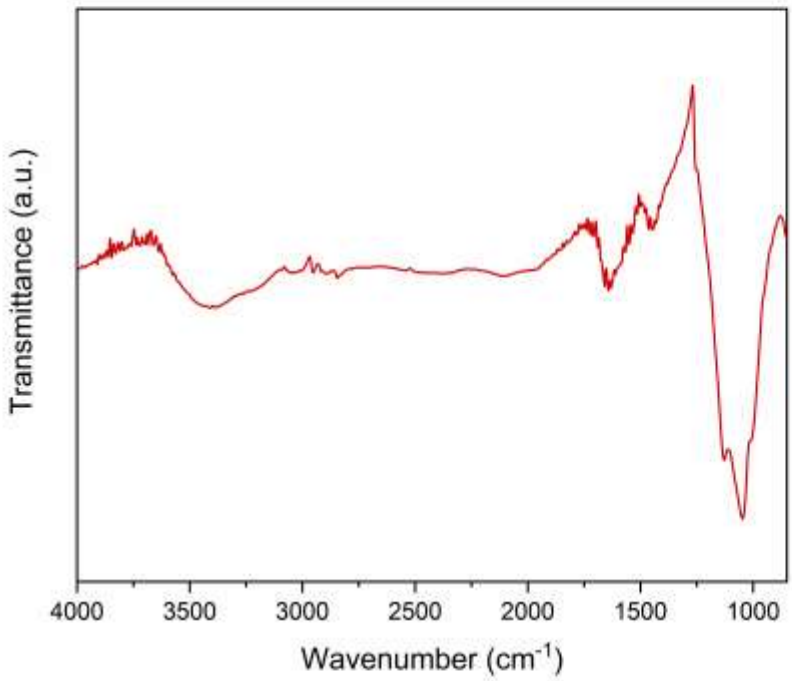


**Figure S13.** FT-IR spectrum of the carbon dots.

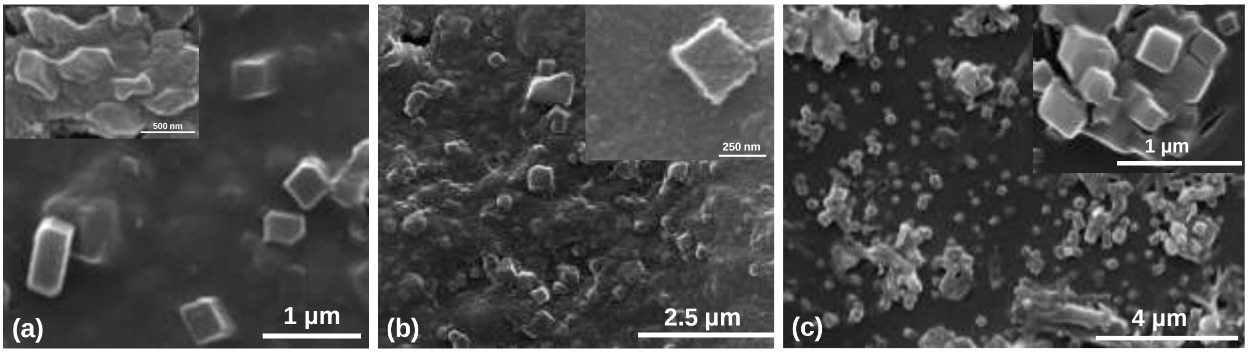


**Figure S14.** Scanning electron microscopic images of the carbon dot embedded host-guest complexes of (a) $D_1AD_1{\subset}CB$; (b) $D_2AD_2{\subset}CB$ and (c) $D_3AD_3{\subset}CB$.

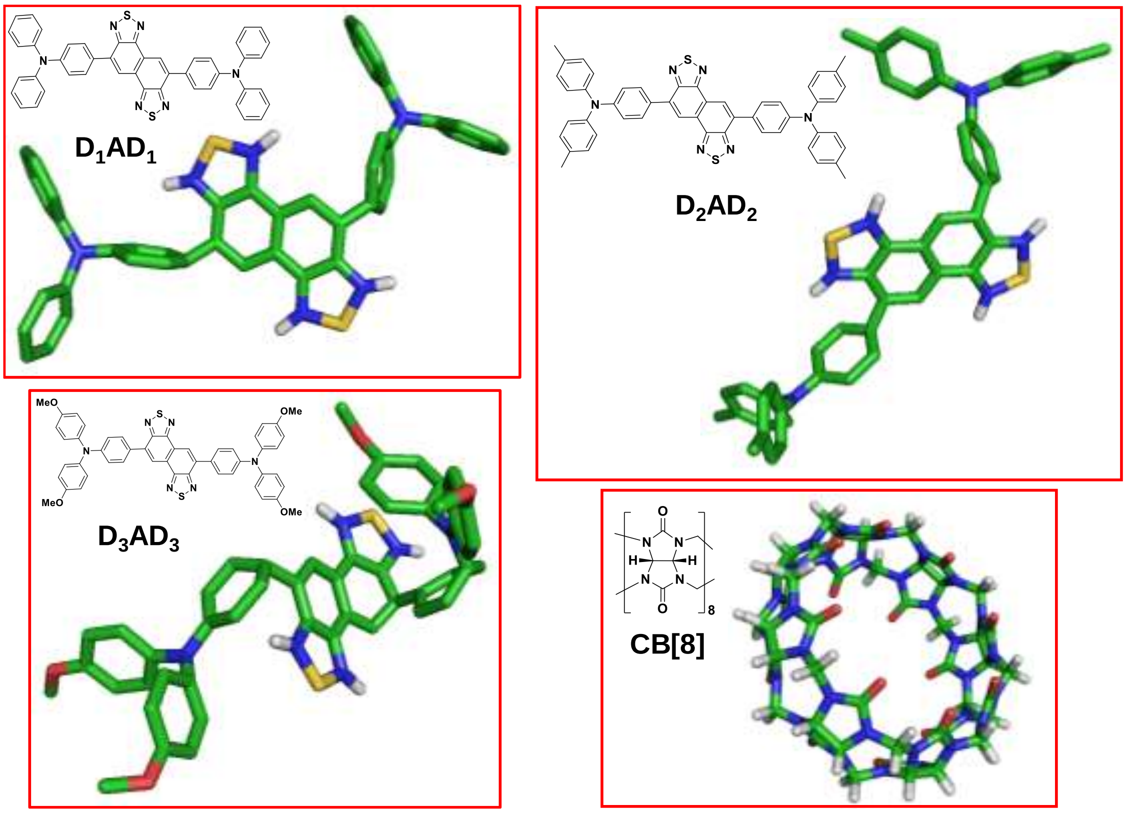


**Figure S15.** Energy minimized structures of $D_1AD_1$, $D_2AD_2$, $D_3AD_3$ and cucurbit[8]uril.

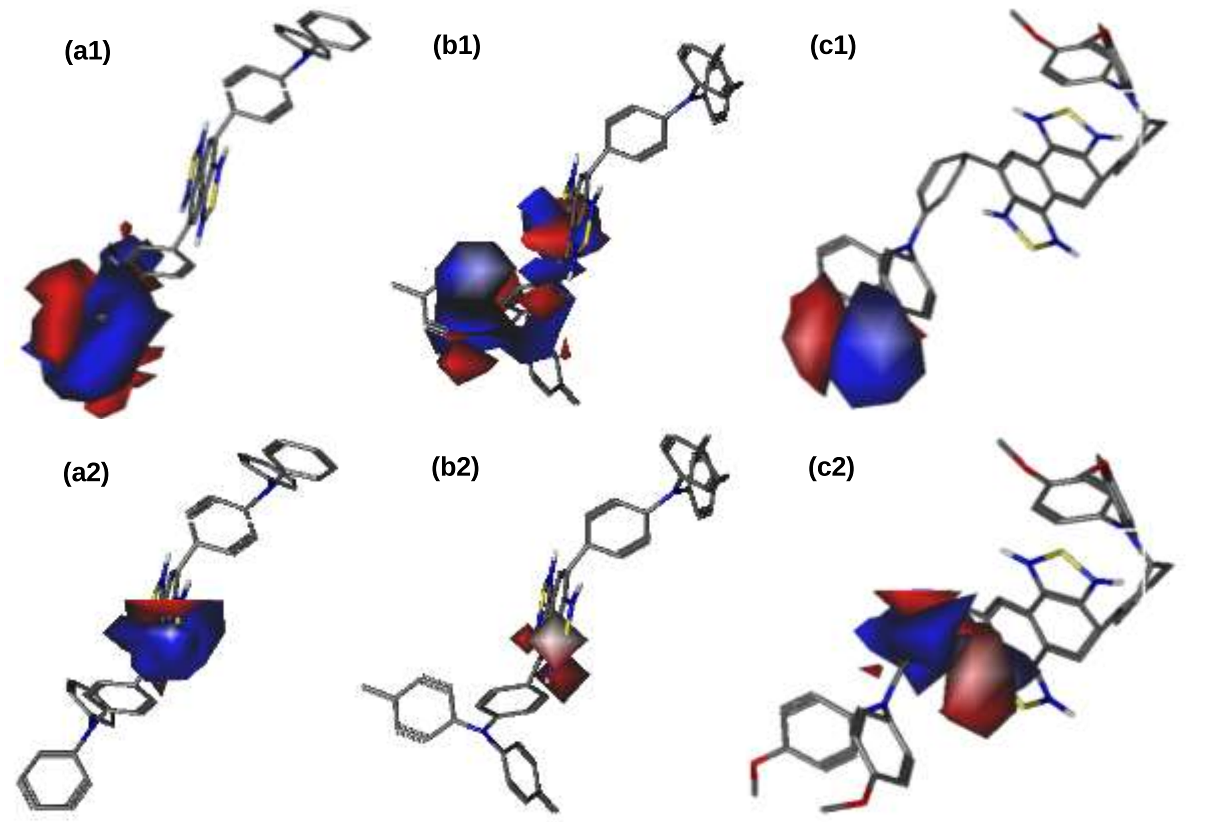


**Figure S16.** Pictorial diagram of (a1,b1,c1) HOMO and (a2,b2,c2) LUMO of the energy minimized structures of (a1,a2) $D_1AD_1$, (b1,b2) $D_2AD_2$ and (c1,c2) $D_3AD_3$.

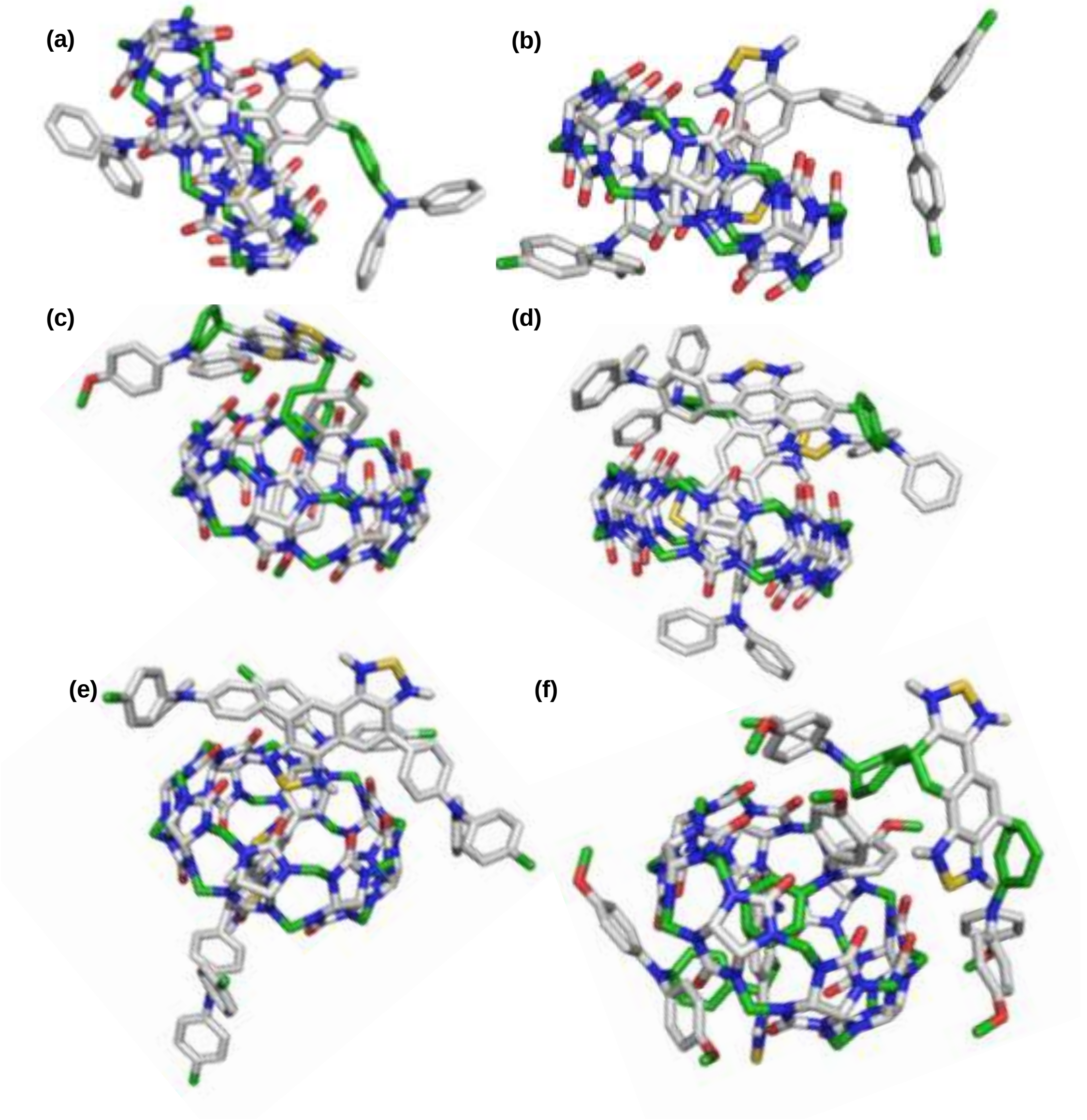


**Figure S17.** The most favorable docked geometries of 1:1 host-guest complexes between cucurbit[8]uril and the NIR-II dyes, *i.e.* (a) $D_1AD_1$; (b) $D_2AD_2$ and (c) $D_3AD_3$. The favored docked geometries for the 1:2 complexes between cucurbit[8]uril and the NIR-II dyes, *i.e.* (d) $D_1AD_1$; (e) $D_2AD_2$ and (f) $D_3AD_3$ were also shown.

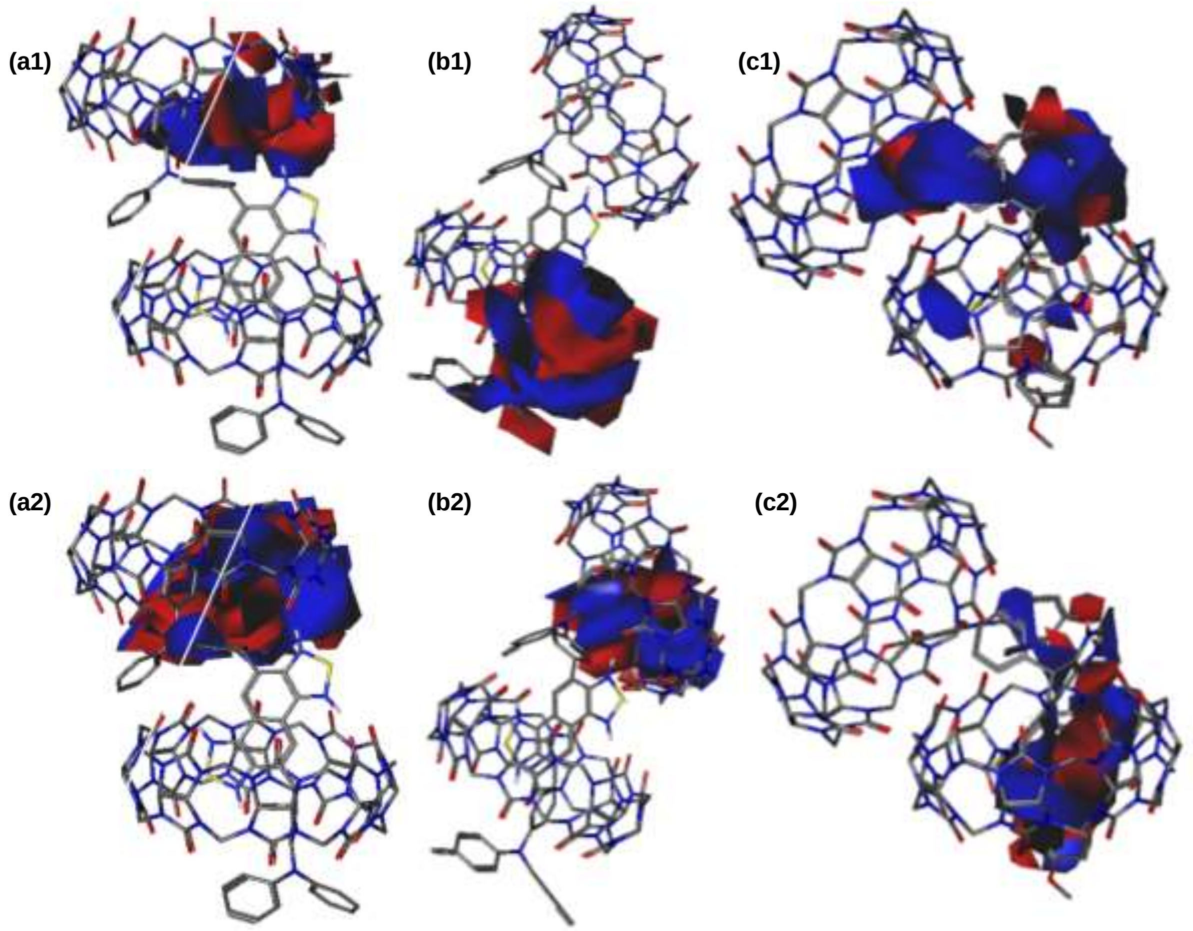


**Figure S18.** Pictorial diagram of (a1,b1,c1) HOMO and (a2,b2,c2) LUMO for the favored docked geometries of 1:2 host-guest complexes between the NIR-II dyes and cucurbit[8]uril respectively, *i.e.*, (a1,a2) $D_1AD_1$, (b1,b2) $D_2AD_2$ and (c1,c2) $D_3AD_3$.

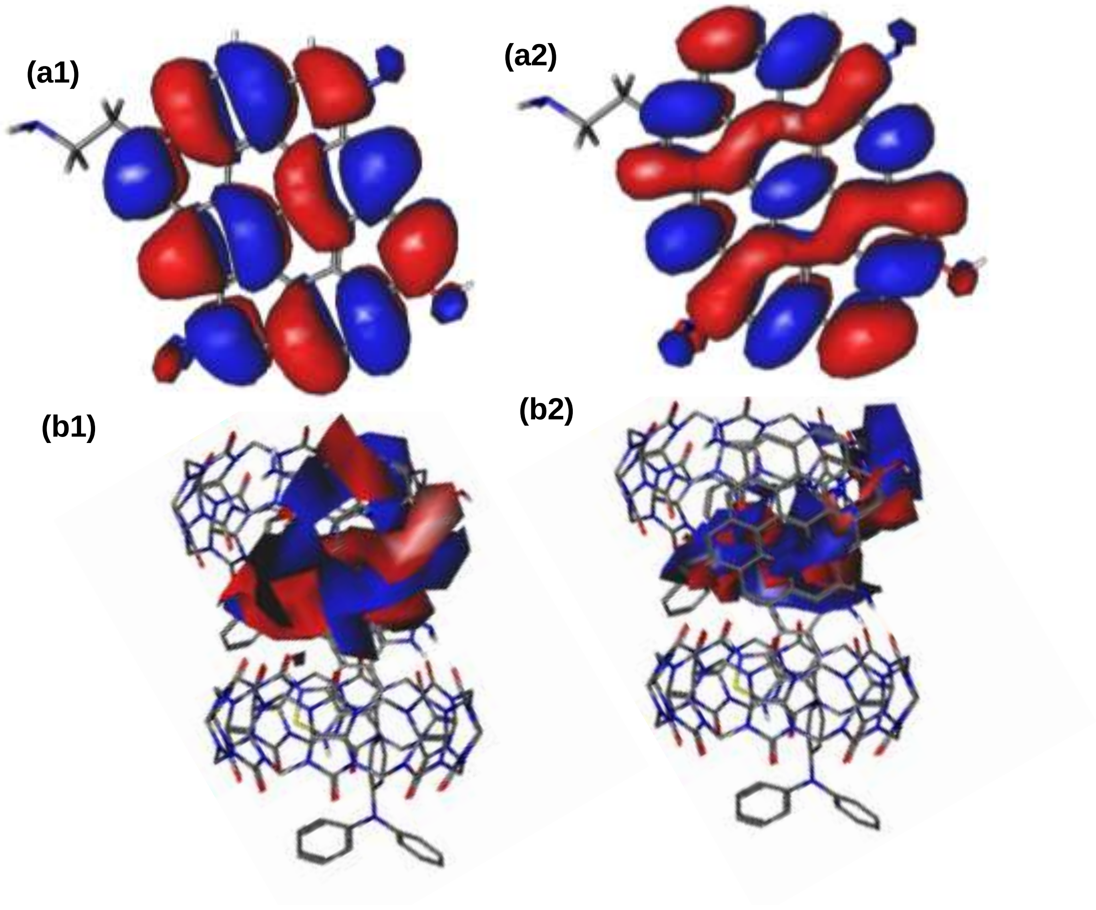


**Figure S19.** Pictorial diagram of (a1,b1) HOMO and (a2,b2) LUMO for the energy minimized structure of (a1,a2) model red carbon dot and (b1,b2) favored docked geometry of the supramolecularly assembled carbon dot and 2:1 host-guest complex between cucurbit[8]uril and $D_1AD_1$ respectively.

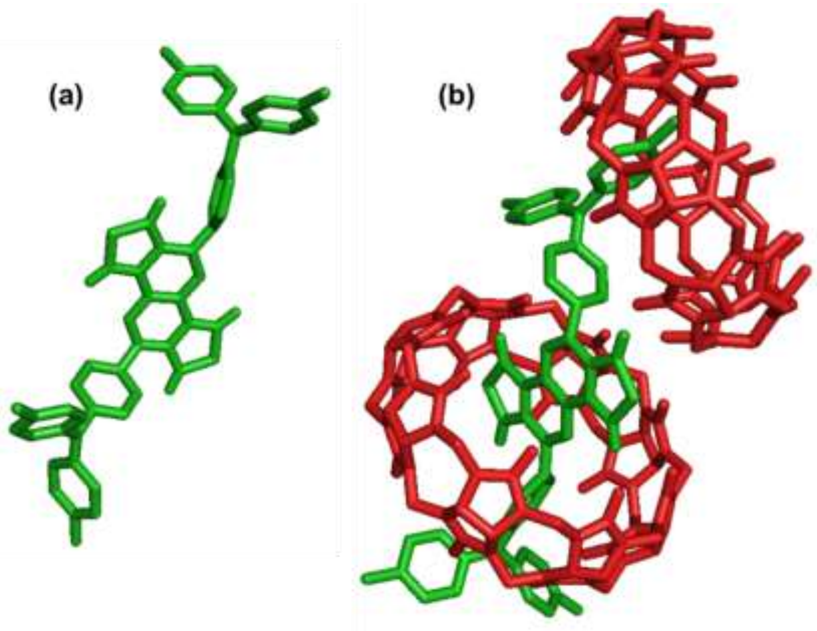


**Figure S20.** Representation of $D_2AD_2$ in (a) free state and (b) inclusion complexed state with CB[8] molecules. The dye is shown in green color and CB[8] molecules in red color.

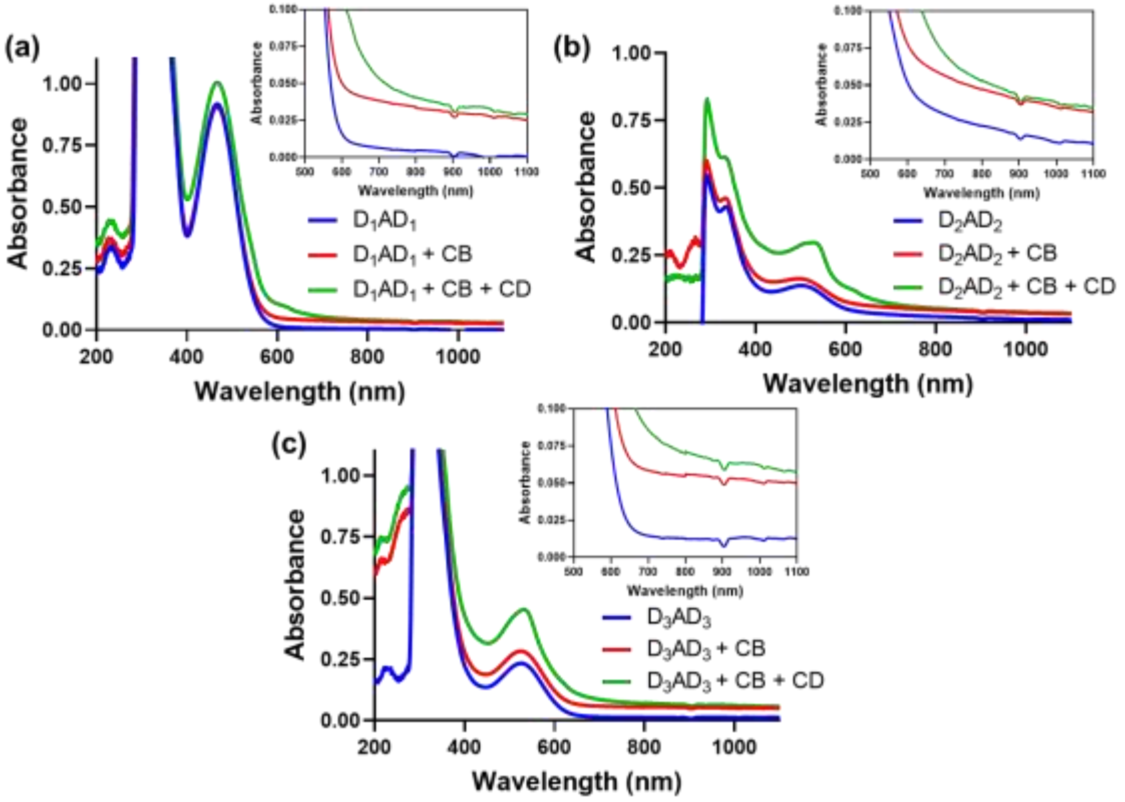


**Figure S21.** UV-Visible spectra of the DAD systems before and after the addition of CB and rCD. The CB and rCD conjugates with $D_1AD_1$, $D_2AD_2$ and $D_3AD_3$ were shown in a, b and c respectively. Inset shows the zoom-in region from 500 to 1100 nm.

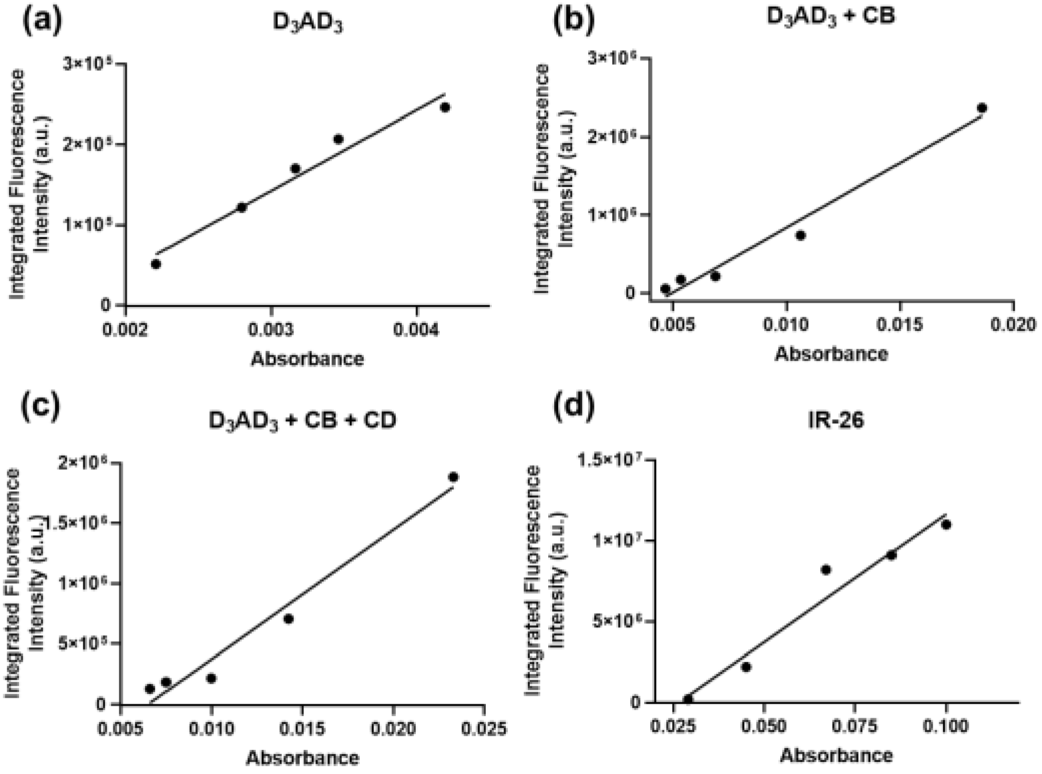


**Figure S22.** Plot of integrated fluorescence intensities for (a) $D_3AD_3$, (b) $D_3AD_3$ + CB, (c) $D_3AD_3$ + CB+ rCD and (d) reference near-infrared fluorophore, IR-26, at five different concentrations. The slopes of the linear fits were compared to calculate the quantum yield of the conjugates. The quantum yield of IR-26 dye was referenced as 0.5%.

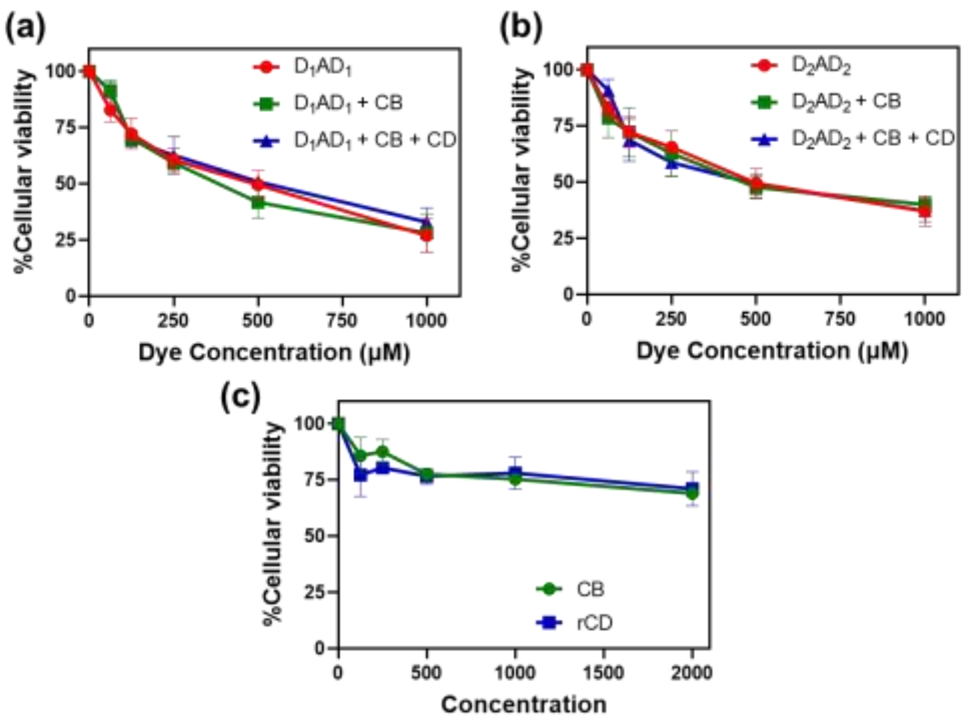


**Figure S23.** Comparative cellular viability of (a) $D_1AD_1$ and (b) $D_2AD_2$ conjugates before and after the addition of CB[8] and rCD in HEK293 cells. (c) The cellular toxicity of CB and rCD was also shown at different concentrations. The concentrations of CB and rCD has been represented in μM and μg/mL respectively.

**Table S1.** Comparative average binding energies for the host-guest complexes formed between CB[8] and NIR-II dyes, *i.e.* $D_1AD_1$, $D_2AD_2$ and $D_3AD_3$.

| **Compounds** | **Ratio** | **Average binding energy (kcal/mol))** | **Number of clusters among 25 different docked geometries** |
|---|---|---|---|
| $D_1AD_1$:CB[8] | 1:1 | -5.168 | 15 |
| $D_2AD_2$:CB[8] | 1:1 | -5.164 | 9 |
| $D_3AD_3$:CB[8] | 1:1 | -4.3024 | 23 |
| $D_1AD_1$:CB[8] | 2:1 | -3.5532 | 15 |
| $D_2AD_2$:CB[8] | 2:1 | +22.0684 | 10 |
| $D_3AD_3$:CB[8] | 2:1 | +5.1144 | 22 |
| $D_1AD_1$:CB[8] | 1:2 | -5.624 | 1 |
| $D_2AD_2$:CB[8] | 1:2 | -5.7988 | 3 |
| $D_3AD_3$:CB[8] | 1:2 | -5.1108 | 3 |

**Table S2.** Clustering histogram for the 1:2 host-guest complexes between NIR-II dyes, *i.e.* $D_1AD_1$, $D_2AD_2$ and $D_3AD_3$, and cucurbit[8]uril with their average binding energy.

CLUSTERING HISTOGRAM

**$D_1AD_1$:CB[8] = 1:2**

| Clus -ter Rank | Lowest Binding Energy | Run | Mean Binding Energy | Num in Clus | Histogram 5 10 15 20 25 30 35 |
|---|---|---|---|---|---|
| 1 | -5.66 | 5 | -5.63 | 25 | ######################### |

Average Energy of Binding = -5.624 kcal/mol

CLUSTERING HISTOGRAM

**$D_2AD_2$:CB[8] = 1:2**

| Clus -ter Rank | Lowest Binding Energy | Run | Mean Binding Energy | Num in Clus | Histogram 5 10 15 20 25 30 35 |
|---|---|---|---|---|---|
| 1 | -5.81 | 21 | -5.81 | 10 | ########## |
| 2 | -5.80 | 20 | -5.80 | 12 | ############ |
| 3 | -5.79 | 1 | -5.78 | 3 | ### |

Average Energy of Binding = -5.7988 kcal/mol

CLUSTERING HISTOGRAM

**$D_3AD_3$:CB[8] = 1:2**

| Clus -ter Rank | Lowest Binding Energy | Run | Mean Binding Energy | Num in Clus | Histogram 5 10 15 20 25 30 35 |
|---|---|---|---|---|---|
| 1 | -5.22 | 16 | -5.20 | 19 | ################### |
| 2 | -4.90 | 12 | -4.85 | 5 | ##### |
| 3 | -4.78 | 18 | -4.78 | 1 | # |

Average Energy of Binding = -5.1108 kcal/mol

**Table S3.** Clustering histogram for the supramolecularly assembled carbon dot embedded 1:2 host-guest complexes between the NIR-II dyes, *i.e.* $D_1AD_1$, $D_2AD_2$ and $D_3AD_3$, and cucurbit[8]uril with their average binding energies.

CLUSTERING HISTOGRAM

**$D_1AD_1$:CB[8]:rCD = 1:2:1**

| Clus -ter Rank | Lowest Binding Energy | Run | Mean Binding Energy | Num in Clus | Histogram (5 10 15 20 25 30 35) |
|---|---|---|---|---|---|
| 1 | -8.45 | 19 | -8.34 | 9 | ######### |
| 2 | -8.35 | 21 | -8.11 | 3 | ### |
| 3 | -7.95 | 25 | -7.83 | 3 | ### |
| 4 | -7.81 | 7 | -7.81 | 1 | # |
| 5 | -7.59 | 15 | -7.56 | 2 | ## |
| 6 | -7.53 | 6 | -7.46 | 5 | ##### |
| 7 | -7.24 | 17 | -7.24 | 1 | # |
| 8 | -5.95 | 23 | -5.95 | 1 | # |

Average Energy of Binding = -7.85 kcal/mol

CLUSTERING HISTOGRAM

**$D_2AD_2$:CB[8]:rCD = 1:2:1**

| Clus -ter Rank | Lowest Binding Energy | Run | Mean Binding Energy | Num in Clus | Histogram (5 10 15 20 25 30 35) |
|---|---|---|---|---|---|
| 1 | -8.50 | 22 | -8.35 | 17 | ################# |
| 2 | -7.85 | 1 | -7.80 | 2 | ## |
| 3 | -7.60 | 12 | -7.60 | 1 | # |
| 4 | -7.60 | 4 | -7.60 | 1 | # |
| 5 | -7.39 | 5 | -7.38 | 3 | ### |
| 6 | -7.12 | 8 | -7.12 | 1 | # |

Average Energy of Binding = -8.078 kcal/mol

CLUSTERING HISTOGRAM

**$D_3AD_3$:CB[8]:rCD = 1:2:1**

| Clus -ter Rank | Lowest Binding Energy | Run | Mean Binding Energy | Num in Clus | Histogram (5 10 15 20 25 30 35) |
|---|---|---|---|---|---|
| 1 | -8.15 | 19 | -8.09 | 10 | ########## |
| 2 | -7.92 | 14 | -7.83 | 12 | ############ |
| 3 | -7.71 | 21 | -7.68 | 2 | ## |
| 4 | -6.91 | 24 | -6.91 | 1 | # |

Average Energy of Binding = -7.886 kcal/mol

## 3. References.


[1] F. Ostadhossein, D. Sar, I. Tripathi, J. Soares, E. E. Remsen, D. Pan, *ACS Appl. Mater. Interfaces* **2020**, *12*, 10183-10192.

[2] J. Walton, P. Wincott, N. Fairley, A. Carrick, Peak Fitting with CasaXPS: A Casa Pocket Book. 2010.

[3] M. W. Schmidt, K. K. Baldridge, J. A. Boatz, S. T. Elbert, M. S. Gordon, J. H. Jensen, S. Koseki, N. Matsunaga, K. A. Nguyen, S. Su, T. L. Windus, M. Dupuis, J. A. Montgomery Jr, *J. Comput. Chem.* **1993**, *14*, 1347–1363.

[4] G. M. Morris, R. Huey, W. Lindstrom, M. F. Sanner, R. K. Belew, D. S. Goodsell, A. J. Olson, *J. Comput. Chem*. **2009**, *16,* 2785-2791.

[5] I. Srivastava, P. Moitra, D. Sar, K. Wang, M. Alafeef, J. Scott, D. Pan, *Nanoscale* **2021**, *13*, 16288.

[6] I. Srivastava, P. Moitra, M. Fayyaz, S. Pandit, T. L. Kampert, P. Fathi, H. R. Alanagh, K. Dighe, M. Alafeef, K. Vuong, M. Jabeen, S. Nie, J. Irudayaraj, D. Pan, *ACS Applied Materials & Interfaces* **2021**, *13*, 59747.

[7] A. L. Antaris, H. Chen, K. Cheng, Y. Sun, G. Hong, C. Qu, S. Diao, Z. Deng, X. Hu, B. Zhang, X. Zhang, O. K. Yaghi, Z. R. Alamparambil, X. Hong, Z. Cheng, H. Dai, *Nature Materials* **2016**, *15*, 235.

[8] F. Ostadhossein, P. Moitra, N. Gunaseelan, M. Nelappana, C. Lowe, M. Moghiseh, A. Butler, N. Deruiter, H. Mandalika, I. Tripathi, S. K. Misra, D. Pan, *Nanoscale Horizons* **2022**, DOI 10.1039/D1NH00626F.
